# Cut-Dependent Topology Optimization for Enhancing Shear-Mode Purity in Lithium Niobate Wafers

**DOI:** 10.3390/s26144443

**Published:** 2026-07-13

**Authors:** Jun Zhou, Ning Hu, Weifeng Yuan, Hui Hu, Kaiyan Huang, Jishuo Wang

**Affiliations:** 1 School of Mechanical Engineering, Hebei University of Technology, Tianjin 300401, China; junez@swust.edu.cn; 2 Key Laboratory of Testing Technology for Manufacturing Process MOE, Southwest University of Science and Technology, Mianyang 621010, China; yuanweifeng@swust.edu.cn (W.Y.); fayhu@swust.edu.cn (H.H.); huangkaiyan@swust.edu.cn (K.H.); jswang@swust.edu.cn (J.W.); 3 Sichuan Electronic and Mechanic Vocational College, Mianyang 621023, China; 4 School of Mechanical Engineering, Xihua University, Chengdu 610039, China; 5 Henan Key Laboratory of Underwater Intelligent Equipment, The 713th Research Institute of China State Shipbuilding Corporation Limited, Zhengzhou 450015, China

**Keywords:** lithium niobate wafer, topology optimization, shear-mode purity, piezoelectric anisotropy, crystal-cut dependence, electrode pattern design

## Abstract

We present a topology-optimization methodology for designing single-sided, tri-state electrode patterns (+V, 0, electrode-free) that maximize shear-mode purity in lithium niobate (LiNbO_3_) wafers. The framework combines a complex-Hermitian adjoint sensitivity formulation based on Wirtinger calculus with a coarse–fine design-mesh decomposition and Heaviside projection, and treats the bottom-face electrical boundary condition as an explicit design variable. Applying the method to five crystal cuts (X, Y, Z, 41°Y, 128°Y) over 3.30–4.10 MHz under grounded and single-sided configurations, we find that the optimal boundary condition is jointly determined by crystal cut and frequency through the rotated piezoelectric tensor and that topology optimization improves purity by up to 28.8 percentage points when the baseline is poorly matched but can be counterproductive when it is already optimal (Z-cut). We distil these behaviors into a three-regime taxonomy that predicts, a priori, when optimization is worthwhile. The result is a reusable design methodology, together with per-cut design rules, for shear-mode LiNbO_3_ transducers.

## 1. Introduction

Lithium niobate (LiNbO_3_) is one of the most widely used single-crystal piezoelectric materials for high-frequency ultrasonic and acoustic-wave devices, owing to its large electromechanical coupling coefficient and low intrinsic loss, and the ability of different crystal cuts to selectively excite distinct acoustic modes [1]. Among these modes, shear-mode transducers, which generate horizontally polarized particle motion perpendicular to the direction of wave propagation, are of particular interest for applications such as bulk acoustic wave (BAW) resonators in radio-frequency front ends [2,3], shear-horizontal surface acoustic wave (SH-SAW) sensors for biosensing in liquid environments, where compressional waves are strongly attenuated [4,5,6,7], and thickness-shear-mode (TSM) sensors [8,9], including quartz crystal microbalances. In these applications, the purity of the excited shear mode, defined as the fraction of stored elastic energy associated with shear deformation relative to longitudinal compression, directly determines the device selectivity, signal-to-noise ratio, and robustness against spurious modes.

Two design choices play a dominant role in determining shear-mode purity in a LiNbO_3_ wafer: the crystal cut, namely, the orientation of the wafer normal with respect to the crystallographic axes, and the electrode geometry on the wafer surfaces [10,11]. Crystal-cut selection has been studied extensively since the 1970s, with X-cut, 41°Y-cut, 36°Y-cut, and 128°Y-cut LiNbO_3_ shown to preferentially excite different families of shear waves [12,13,14,15]. Electrode design, in turn, has traditionally relied on canonical geometries such as full-plate or bipolar (half-and-half) patterns. Both paradigms are geometrically uniform and are typically selected on a cut-by-cut, largely empirical basis.

In recent decades, topology optimization [16,17,18,19,20] has emerged as a powerful framework for generalizing these classical electrode designs. Rather than restricting the electrode to a prescribed configuration, such as a uniform plate or an interdigital pattern, topology optimization treats the local electrode density at each surface point as a continuous design variable and iteratively redistributes the conductive domain to maximize a prescribed objective under specified constraints [21,22,23,24]. When adapted to piezoelectric systems, this approach has produced electrode patterns with non-intuitive geometric features, including fragmented patches, asymmetric layouts, and graded transitions, that can systematically outperform conventional uniform designs in applications ranging from energy harvesting to vibration suppression and acoustic-wave focusing [25,26].

Despite these advances, two important gaps remain in the literature on topology optimization for piezoelectric shear-mode transducers. First, prior studies have predominantly focused on double-sided electrode configurations, in which patterned electrodes are placed on both the top and bottom faces of the wafer [27,28]. Single-sided configurations, in which only the top face is patterned while the bottom face is either covered by a continuous grounded plane or left electrically free, are of substantial practical interest because they reduce fabrication complexity by requiring only one lithography step and facilitate integration with planar substrates such as glass or silicon wafers [29]. However, their treatment within topology optimization remains comparatively underdeveloped. Second, and more importantly, in the limited number of studies that consider single-sided geometries, the bottom-face electrical boundary condition is usually fixed during the design stage, typically as a grounded plane or electrode-free, and treated as a prescribed fabrication constraint rather than an active design dimension [30,31].

As demonstrated in this work, however, the choice between a grounded and an electrically free bottom face fundamentally alters the attainable shear-mode purity, and the optimal choice is cut-dependent, being governed by the structure of the rotated piezoelectric tensor. In this paper we present a complete topology-optimization framework for ternary single-sided electrode patterns (+V, 0, and electrode-free) on 10 × 10 × 0.5 mm LiNbO_3_ wafers, with the bottom-face boundary configuration treated explicitly as a design variable. The wafer dimensions place the fundamental thickness-shear resonance within the conventional ultrasonic non-destructive testing (NDT) band [32,33,34] ($f_{1}=v_{s}/2t$ ≈ 4.0 MHz for t = 0.5 mm); the 3.30–4.10 MHz band examined in Section 4 spans about ±10% around this resonance, representative of NDT shear-transducer bandwidths [35]. The 20:1 lateral-to-thickness aspect ratio ensures the thickness resonance dominates over lateral standing waves, consistent with classical thickness-mode theory [36], and wafers of these dimensions are commercially available for all five cuts studied.

These dimensions also position the study as a wafer-level foundation for a forthcoming multi-mode ultrasonic NDT investigation [37,38,39,40,41], which will require high purity across multiple shear-mode families over a broader band. The methodological framework comprises a complex-Hermitian adjoint sensitivity formulation based on Wirtinger calculus [42]—with explicit distinction between the complex transpose and the Hermitian transpose, essential under structural damping—together with a coarse–fine design-mesh decomposition and Heaviside projection for manufacturable two-tone electrode patterns.

In particular, we report the counterintuitive case of Z-cut LiNbO_3_ under single-sided drive, where the simple bipolar electrode pattern already saturates the wafer-level physical purity limit at 97.15%, and topology optimization can instead reduce purity by up to 43 percentage points. These results lead to a three-regime classification of baseline quality. This challenges the implicit assumption that topology optimization is universally advantageous for piezoelectric electrode design.

More broadly, the use of geometry and electrical boundary conditions to control elastic-wave modes connects this work to engineered acoustic and phononic structures. In piezoelectric phononic-crystal plates, the elastic bandgap and mode content can be tuned by the electrode boundary condition—grounded, short-circuited or floating—and by the electrode distribution [43], and topology optimization has been used to shape phononic bandgaps in piezocomposite media [44]. While those studies concern periodic wave-propagation media rather than the single-wafer thickness-resonance regime considered here, they share the principle exploited in this work: electrode geometry and bottom-face electrical boundary conditions are active design levers for selecting and purifying acoustic modes.

Beyond ultrasonic NDT and sensing, high-purity shear modes in LiNbO_3_ are increasingly important in straintronic magnonic devices, where acoustic waves manipulate spin waves through magnetoelastic coupling [45]; optimizing the electrode pattern to control the strain field, as done here, is therefore directly relevant to efficient electromechanical transduction in such hybrid systems. The strong dependence of the mode profile on the electrical boundary condition also connects to electrodynamic boundary-condition control in layered ferrite–ferroelectric structures, where breaking field symmetry enables nonreciprocal propagation [46]; the optimized single-sided patterns proposed here could serve as the piezoelectric layer in such hybrid heterostructures.

The primary contribution of this work is methodological—a manufacturability-aware, tri-state, single-sided topology-optimization framework with a complex-Hermitian (Wirtinger) adjoint that treats the bottom-face electrical boundary condition as an explicit design variable. Applying this methodology across five LiNbO_3_ cuts yields the secondary contribution: the finding that the optimal bottom-face boundary condition depends jointly on crystal cut and frequency, and a three-regime taxonomy that predicts, a priori, when topology optimization yields significant gains. Together these distinguish the present study from prior work that treated electrode geometry and crystal orientation separately.

We now formalize this problem: Section 2 introduces the rotated-tensor piezoelectric model and the energy-based shear-mode purity objective that the optimization targets, and Section 3, Section 4 and Section 5 present the numerical formulation, the results across all cut–frequency–boundary combinations, and the conclusions.

## 2. Theoretical Framework

This section establishes the continuous-form physical theory underlying the present study, independent of any particular numerical discretization or implementation strategy. We first formulate the constitutive relations of linear piezoelectricity, introduce the crystal-cut rotation formalism that connects the crystallographic and laboratory coordinate frames, and derive the frequency-domain weak form in the presence of structural damping. We then define the three physically distinct bottom-face electrical boundary configurations whose comparison is central to this work. Finally, we formalize shear-mode purity as the physical objective to be maximized in the subsequent topology-optimization framework.

### 2.1. Linear Piezoelectric Constitutive Relations

We consider a piezoelectric wafer occupying the rectangular domain $\Omega =[0,L_{x}]\times [0,L_{y}]\times [0,L_{z}]$ with $L_{x}=L_{y}=10$ mm and $L_{z}=0.5$ mm. The wafer is composed of single-crystal lithium niobate (LiNbO_3_), which belongs to the trigonal point group 3 m. We adopt small-strain linear piezoelectric theory, with the mechanical displacement field $u(x,t)=(u_{1},u_{2},u_{3}{)}^{⊤}$ and electrostatic potential φ(x,t) taken as the primary unknowns. The corresponding kinematic quantities are the symmetric strain tensor and the electric field:

(1) $$S_{ij}=\frac{1}{2}(\partial_{i}u_{j}+\partial_{j}u_{i}),E=-\nabla \varphi$$

with the strain expressed in Voigt vector form $S=(S_{11},S_{22},S_{33},2S_{23},2S_{13},2S_{12}{)}^{⊤}$ using the engineering shear–strain convention.

The linear piezoelectric constitutive law couples the mechanical and electrical fields through the standard stress–charge relations:

(2) $$\begin{array}{ll} T & =c^{E}S-e^{⊤}E\\ D & =e\;S+\varepsilon^{S}E \end{array}$$

where $T\in R^{6}$ denotes the stress vector in Voigt notation, $D\in R^{3}$ is the electric displacement vector, $c^{E}\in R^{6\times 6}$ is the elastic stiffness tensor at constant electric field, $e\in R^{3\times 6}$ is the piezoelectric stress tensor, and $\varepsilon^{S}\in R^{3\times 3}$ is the permittivity tensor at constant strain.

The governing equations consist of the elastodynamic momentum balance and Gauss’s law for free charge under the electrostatic approximation:

(3) $$\rho \;{\ddot{u}}_{i}=\partial_{j}T_{ij},\nabla \cdot D=0$$

with ρ denoting the mass density. The electrostatic approximation is justified because, at MHz frequencies, the acoustic wavelength is many orders of magnitude shorter than the corresponding electromagnetic wavelength. Throughout this work, all field quantities are evaluated at a single excitation angular frequency ω=2πf, such that $u(x,t)=Re[\tilde{u}(x)e^{i\omega t}]$, with an analogous harmonic representation for φ.

### 2.2. Crystal-Cut Rotation and Rotated Material Tensors

The material tensor values of LiNbO_3_ in the Z-frame, corresponding to the crystallographic principal-axis frame, are taken from [47]: $c_{11}^{E}=203.0$ GPa, $c_{12}^{E}=57.3$ GPa, $c_{13}^{E}=75.2$ GPa, $c_{14}^{E}=8.5$ GPa, $c_{33}^{E}=242.4$ GPa and $c_{44}^{E}=59.5$ GPa. The relation $c_{66}^{E}=(c_{11}^{E}-c_{12}^{E})/2=72.85$ GPa is imposed by the trigonal 3 m symmetry. The piezoelectric stress tensor is expressed in C⋅m^−2^,

(4) $$e^{Z}=\left(\begin{array}{cccccc} 0 & 0 & 0 & 0 & 3.7 & -2.5\\ -2.5 & 2.5 & 0 & 3.7 & 0 & 0\\ 0.23 & 0.23 & 1.33 & 0 & 0 & 0 \end{array}\right)$$

and the permittivity tensor is $\varepsilon^{S}=diag(44,\;44,\;27.9)\;\varepsilon_{0}$, where $\varepsilon_{0}$ denotes the vacuum permittivity. The mass density is ρ=4647 kg⋅m^−3^.

For each crystal cut considered in this study, namely, X-, Y-, Z-, 41°Y- and 128°Y-cut LiNbO_3_, the rotation matrix R∈SO(3) from the crystal frame to the laboratory frame is constructed such that the wafer normal is aligned with the laboratory z-axis. Specifically, the X-cut uses $R=R_{y}(+\pi /2)$, the Y-cut uses $R=R_{x}(-\pi /2)$, the Z-cut uses R=I, and a generic θ°Y rotated cut uses $R_{x}(-\pi /2+\theta \pi /180)$. The rotated material tensors are then obtained through the corresponding tensor-transformation relations:

(5) $$c^{\prime E}=Mc^{E}M^{⊤},\;e^{\prime }=R\;e\;M^{⊤},\;\varepsilon^{\prime \;S}=R\;\varepsilon^{S}R^{⊤}$$

where $M\in R^{6\times 6}$ denotes the engineering-strain Bond matrix associated with the orthogonal rotation R.

A subtle but important implication of the engineering shear–strain convention should be emphasized. Although R is orthogonal, the corresponding Bond matrix M for engineering strain is not, in general, $M^{⊤}M\neq I$. The elastic-energy quadratic form, $\frac{1}{2}T^{⊤}S$, remains invariant under rotation because the corresponding stress-transformation matrix is $N=(M^{-1}{)}^{⊤}$. Consequently, $T^{\prime ⊤}S^{\prime }=T^{⊤}N^{⊤}MS=T^{⊤}S$**,** since $N^{⊤}M=I$. This distinction is essential for the numerical verification of tensor-rotation routines.

### 2.3. Frequency-Domain Weak Form with Structural Damping

Material attenuation is introduced through a structural loss factor η=2ζ by applying the complex stiffness substitution $c^{\prime \;E}\to (1+i\eta )\;c^{\prime \;E}$. We set ζ=0.02 for both the grounded and single-sided configurations reported throughout this work, and ζ=0.05 only for the alternative floating (open-circuit) implementation. These correspond to effective quality factors Q≈25 and Q≈10, representative of a mounted, backed shear-mode NDT transducer rather than the far higher intrinsic Q of bulk LiNbO_3_ (unbacked thickness-shear resonators reach Q≈3500 at ~6 MHz [48]). Because shear-mode purity is an energy ratio, it is essentially independent of the absolute loss level; the grounded (A) versus single-sided (C) comparison is therefore performed at identical damping.

The frequency-domain weak form is stated as follows: find $(\tilde{u},\tilde{\varphi })\in V$ such that, for all admissible test functions (δu,δφ),

(6) $$\begin{array}{ll} \int_{\Omega }\delta S^{⊤}(1+i\eta )\;c^{\prime \;E}\;\tilde{S}\;dV & +\int_{\Omega }\delta S^{⊤}e^{\prime ⊤}\;\nabla \tilde{\varphi }\;dV-\omega^{2}\int_{\Omega }\rho \;\delta u^{⊤}\tilde{u}\;dV=0,\\ \int_{\Omega }\delta (\nabla \varphi {)}^{⊤}e^{\prime }\;\tilde{S}\;dV & -\int_{\Omega }\delta (\nabla \varphi {)}^{⊤}\varepsilon^{\prime \;S}\nabla \tilde{\varphi }\;dV=0. \end{array}$$

The coupled electromechanical variational equations are satisfied. This formulation corresponds to the symmetric Allik–Hughes form [49]. The second equation is globally negated so that the resulting block operator is complex-symmetric, $A^{⊤}=A$, rather than Hermitian, $A^{H}={\bar{A}}^{⊤}\neq A$. This distinction has direct consequences for the adjoint sensitivity formulation and constitutes one of the most frequently overlooked sources of error in damped piezoelectric topology optimization.

### 2.4. Bottom-Face Boundary Configurations

A central physical observation motivating this work is that the bottom-face electrical boundary condition should be treated as a design choice rather than as a fixed fabrication detail, and that the optimal choice is crystal-cut dependent. We consider three physically distinct configurations, distinguished by their bottom-face electrical treatment and the resulting top-face driving scheme. Although all three configurations are equivalent up to a constant offset in φ, so that the choice of ground reference has no intrinsic physical content, they impose qualitatively different electric-field topologies and therefore couple differently to the rotated piezoelectric tensor associated with each crystal cut.

#### 2.4.1. Configuration A: Grounded Plane (Sandwich Electrode)

In Configuration A, a continuous metallic ground plane covers the entire bottom face and enforces the corresponding Dirichlet condition.

(7) $$\varphi (x,y,0)=0\;\forall \;(x,y)\in [0,L_{x}]\times [0,L_{y}]$$

The top-face drive is symmetric, with the positive-polarity electrodes prescribed as $V_{+}=+V_{0}/2$ and the negative-polarity electrodes prescribed as $V_{-}=-V_{0}/2$. This geometry corresponds to a standard sandwich-electrode capacitor and produces a strong through-thickness electric field $E_{z}$, wherever a top electrode is present. It therefore represents the canonical configuration for shear modes excited through $E_{z}$-coupled piezoelectric tensor components, such as ${e^{\prime }}_{35}$ for X-cut LiNbO_3_.

#### 2.4.2. Configuration B: Floating with Symmetric Drive

In Configuration B, no metallization is present on the bottom face, which is therefore electrically free. The physically appropriate boundary condition is the natural Neumann condition $D_{n}=0$, corresponding to zero normal electric displacement. This condition is imposed implicitly by the weak form in Equation (6) on any boundary not subject to a Dirichlet constraint. The top-face drive remains symmetric, with $V_{+}=+V_{0}/2$ and $V_{-}=-V_{0}/2$.

A subtle feature of this configuration is that the resulting boundary-value problem retains an additive null mode, φ→φ+C, for an arbitrary constant C, because only potential differences are physically meaningful. This null mode renders the finite-element system singular and therefore requires regularization, whose numerical implementation is discussed in Section 3.

#### 2.4.3. Configuration C: Single-Sided Drive

As in Configuration B, the bottom face in Configuration C is electrically free. However, unlike Configuration B, the top-face drive is asymmetric, with $V_{+}=V_{0}$ and $V_{-}=0$. In this scheme, the zero-polarity electrode itself serves as the reference, thereby automatically eliminating the additive null mode without requiring artificial regularization. This configuration most directly represents the physical operation of standard single-sided transducers, including interdigital, coplanar, and surface-acoustic-wave devices, in which one electrode set is held at the drive voltage, the other is held at device ground, and the bottom face remains unmetallized.

#### 2.4.4. Equivalence of Configurations B and C

Configurations B and C produce identical electric fields, mechanical displacements, and elastic energies; they differ only in the choice of zero reference for the electric potential. This equivalence can be stated more precisely as follows. Let $\left({\tilde{u}}_{B},\tilde{\varphi_{B}}\right)$ denote the solution under Configuration B with symmetric drive, $\left(V_{+},V_{-})=(+V_{0}/2,-V_{0}/2\right)$, and with the spatial-mean condition $\int_{bottom}\tilde{\varphi_{B}}\;dA=0$, which uniquely fixes the additive constant. Define $\tilde{\varphi_{C}}:=\tilde{\varphi_{B}}+V_{0}/2$. Then, $\nabla \tilde{\varphi_{C}}=\nabla \tilde{\varphi_{B}}$, and therefore E,D,T, and $\tilde{u}$ are identical. The top-face Dirichlet conditions transform from $\tilde{\varphi }=\pm V_{0}/2$ to $\tilde{\varphi_{C}}=V_{0}$ or 0, exactly reproducing the drive condition of configuration C. The spatial-mean condition becomes $\int_{bottom}(\tilde{\varphi_{C}}-V_{0}/2)\;dA=0$, which is equivalent to a uniformly shifted potential reference.

Configuration C therefore solves the same field problem, with the additive constant fixed by the zero-polarity electrode rather than by a global anchoring condition. Consequently, the shear, longitudinal, and total elastic energies—and hence the shear-mode purity defined in Section 2.5—are mathematically identical in exact arithmetic. Numerical differences between Configuration B and C arise only from finite-precision arithmetic and from the specific regularization used to remove the null mode in Configuration B; in our simulations, these differences are bounded by $10^{-4}$ in $\eta_{s}$.

In the remainder of this paper, we use Configuration C as the operational implementation of the floating case, both because it requires no artificial anchoring condition and because it most closely matches the physical realization of practical single-sided transducers. Accordingly, the comparison between the grounded family, represented by Configuration A, and the single-sided family, represented by Configuration C, is retained as the operative configuration pair for Section 4.

### 2.5. Shear-Mode Purity as Physical Objective

The objective of this work is to maximize *shear-mode purity*, defined as the fraction of the total elastic strain energy stored in shear deformations.

(8) $$\eta_{s}(\tilde{u})=\frac{J_{s}}{J_{s}+J_{l}}$$

where the shear and longitudinal energies are

(9) $$J_{s}=\frac{1}{2}\int_{\Omega }\tilde{S}*\cdot {c^{\prime }}_{s}^{E}\tilde{S}\;dV,J_{l}=\frac{1}{2}\int_{\Omega }\tilde{S}*\cdot {c^{\prime }}_{l}^{E}\tilde{S}\;dV$$

with the elastic stiffness partitioned via the Voigt-component projectors

(10) $$P_{l}=diag(1,1,1,0,0,0),\;P_{s}=diag(0,0,0,1,1,1)$$

(11) $${c^{\prime }}_{s}^{E}=P_{s}\;c^{\prime E}\;P_{s},\;{c^{\prime }}_{l}^{E}=P_{l}\;c^{\prime E}\;P_{l}$$

The asterisk in Equation (9) denotes complex conjugation, reflecting the fact that $\tilde{S}$ is, in general, complex-valued in the presence of structural damping.

The shear-mode purity $\eta_{s}\in [0,1]$ is dimensionless and, by construction, independent of the absolute excitation amplitude. It directly quantifies the wafer-level performance metric of practical interest: the fraction of acoustic energy carried by shear modes relative to that associated with longitudinal compression. A higher $\eta_{s}$ corresponds to a transducer with a cleaner modal response, lower spurious-mode content, and, subject to the limitations of the wafer-level analysis discussed in Section 5.2, improved selectivity for downstream applications such as biosensing in liquid environments where compressional waves cannot propagate.

The partition in Equations (10) and (11) discards the shear–longitudinal cross-coupling term $J_{cross}=S^{*T}(P_{l}c^{\prime }P_{S})S$, so that $J_{total}=J_{s}+J_{l}+J_{cross}$. Near thickness-shear resonance, this term is second-order in the small longitudinal strain; evaluated at the optimized designs, it remains below 3% of $J_{s}+J_{l}$ for every cut considered (0.04% for X-cut, 1.3% for 41°Y, 2.9% for 128°Y), shifting $\eta_{s}$ by at most 2.6 percentage points and preserving all comparative rankings. An energy-based ratio is preferred over a displacement-polarization measure because it is coordinate-invariant and requires no arbitrary choice of reference axis in the anisotropic crystal.

Shear-mode purity is adopted here as the sole optimization objective for three reasons. First, it is the primary determinant of mode selectivity and signal-to-noise ratio in shear-wave NDT and sensing—the target application—since spurious longitudinal content directly degrades flaw discrimination. Second, being a dimensionless energy ratio, it is independent of excitation amplitude, absolute loss level, and mesh resolution, which allows different crystal cuts and boundary configurations to be compared on an equal footing; amplitude-based metrics would conflate material response with drive conditions. Third, purity is partially aligned with, rather than orthogonal to, other common figures of merit: a response that is spectrally pure in shear inherently suppresses the unwanted longitudinal content that limits electromechanical coupling and spurious-mode rejection. A full multi-objective treatment—for example, a purity–amplitude trade-off—is discussed as future work in Section 5.2.

## 3. Numerical Implementation

This section translates the continuous model of Section 2 into a discrete optimization problem. The frequency-domain weak form of Equation (6) is discretized with Q1 trilinear hexahedral finite elements on a 40×40×4 mesh over the 10 × 10 × 0.5 mm wafer, yielding a complex-valued coupled piezoelectric system. The element matrices, their assembly, and the degree-of-freedom scaling required to condition the disparate mechanical and electrical unknowns are given in Supplementary Materials, Sections S1 and S2.

### 3.1. Electrode Design Field

The optimization variable is the top-surface electrode layout. Following the tri-state drive of Section 2.4, each point of the top face is a positive electrode (+V_0_), a negative electrode (−V_0_), or electrode-free, represented by a continuous design field f(x, y) ∈ [−1, +1] that the optimizer evolves and that is projected onto the three physical states on output. To guarantee manufacturable, mesh-independent designs, f is defined on a coarse 10 × 10 design grid and interpolated onto the 40 × 40 top-face element grid. This coarse–fine decomposition fixes the minimum electrode feature size at 1 mm—comfortably within standard fabrication limits—and is the reason the optimized patterns reported in Section 4, including the fragmented 128°Y patterns, remain physically realizable. The tri-state Heaviside projection, the density filter, and the coarse–fine interpolation are detailed in Supplementary Materials S3.

### 3.2. Boundary Enforcement and Design Objective

The three bottom-face configurations defined in Section 2.4 (grounded, floating, and single-sided) and the prescribed top-face potentials are imposed weakly through a soft-penalty formulation; its detailed construction, together with the configuration-dependent safeguard that rejects diverging trial steps, is given in Supplementary Materials S4 and S9. The design objective links the physical target of Section 2.5 to the manufacturability requirement above: it maximizes the shear-mode purity $\eta_{s}$ of Equation (8) while two soft regularization terms drive the electrode area fractions toward the balanced bipolar target (w^+^ = w^−^ = 0.5) and penalize excess perimeter.

The discretized optimization objective combines the shear-mode purity defined in Equation (8) with two soft regularization terms. Discretizing (8) on the finite-element mesh,

(12) $$\eta_{s}({\tilde{u}}_{m})=\frac{J_{s}}{J_{s}+J_{l}},\;J_{s}=\frac{1}{2}\;{\tilde{u}}_{m}^{H}K_{s}{\tilde{u}}_{m},\;J_{l}=\frac{1}{2}\;{\tilde{u}}_{m}^{H}K_{l}{\tilde{u}}_{m}$$

with ${\tilde{u}}_{m}\in C^{3N_{node}}$ the mechanical sub-vector of $\tilde{U}$ and the partitioned mechanical stiffness matrices

(13) $$K_{s,l}=Asm[\int_{\Omega_{e}}B_{u}^{⊤}{c^{\prime }}_{s,l}^{E}B_{u}\;dV]$$

assembled from the partitioned elastic stiffness ${c^{\prime }}_{s,l}^{E}$ (Equation (11)). To prevent the optimizer from collapsing onto trivial single-electrode solutions and to enforce manufacturable patterns, we augment $\eta_{s}$ with two soft regularizers:

(14) $$J(f)=\eta_{s}-\lambda_{A}[(\bar{w^{+}}-V_{T}{)}^{2}+(\bar{w^{-}}-V_{T}{)}^{2}]-\lambda_{p}\;\bar{|\nabla \tilde{f}{|}^{2}}$$

with $\bar{w^{\pm }}=(1/N_{cell})\sum_{c}w_{c}^{\pm }$ the mean fractional electrode coverage, $V_{T}=0.45$ a target per-polarity fraction, $\lambda_{A}=0.30$ the area-balance weight, and $\lambda_{p}=0.05$ the perimeter-smoothness weight. The perimeter term uses forward differences on the filtered design field,

(15) $$g_{x}(j,i)=\tilde{f}(j,i+1)-\tilde{f}(j,i),\;g_{y}(j,i)=\tilde{f}(j+1,i)-\tilde{f}(j,i),\;\bar{|\nabla \tilde{f}{|}^{2}}\;=\frac{1}{N}\sum_{j,i}(g_{x}^{2}+g_{y}^{2})$$

A useful property of the forward-difference convention is that the corresponding gradient with respect to f is exactly the standard five-point Laplacian:

(16) $$\frac{\partial }{\partial \tilde{f}(j,i)}\bar{|\nabla \tilde{f}{|}^{2}}=-\frac{2}{N}\;[\Delta_{5}\;\tilde{f}](j,i)$$

with appropriate Neumann-like boundary contributions, simplifying the adjoint chain.

The area term is central to the results of Section 4: because it favors the balanced bipolar layout, it explains why topology optimization can be counterproductive for cuts—notably, Z-cut—whose bipolar baseline is already optimally matched to the rotated tensor.

### 3.3. Sensitivity and Optimization

Gradients of the objective with respect to the design field are obtained from a complex-Hermitian adjoint formulation based on Wirtinger calculus, which correctly distinguishes the complex transpose from the Hermitian transpose—a distinction that becomes essential once structural damping renders the system matrix complex. The full derivation, the backward-propagation chain that maps the adjoint solution to design-cell sensitivities, and its finite-difference verification are given in Supplementary Materials S5 and S8. The design field is updated by a projected-gradient scheme with backtracking line search (move limit Δ_0_ = 0.15, up to 120 outer iterations), embedded in a Heaviside-projection continuation ($\beta_{0}$ = 4 → $\beta_{max}$ = 10) that progressively sharpens the electrode boundaries, with occasional simulated-annealing perturbations to escape shallow local optima. Because this landscape is non-convex, each case is solved from several independent random initializations (multistart) and the best result is retained; the robustness of this procedure is quantified in Section 4.1. The complete algorithm, including the divergence safeguard, is given in Supplementary Materials S9.

The resulting framework is applied in Section 4 to the five crystal cuts (X, Y, Z, 41°Y, 128°Y) and the two bottom-face configurations across the 3.30–4.10 MHz band.

## 4. Results and Discussion

This section presents the simulation results obtained using the computational framework described in Section 3 and discusses their physical and design implications. The discussion is organized around five principal findings: (i) validation against the X-cut bipolar baseline, which serves as an internal consistency check for the proposed framework; (ii) the largest optimization gains observed for crystal cuts whose baseline electrode patterns are mismatched to their dominant coupling pathways; (iii) the cut- and frequency-dependent nature of the optimal bottom-face electrical boundary condition; (iv) the Z-cut counterexample, which reveals practical limits of topology optimization under soft-penalty constraints; and (v) a unified three-regime taxonomy that provides practical design recommendations.

### 4.1. Simulation Setup and Validation

#### 4.1.1. Numerical Experiment

**Figure 1:**
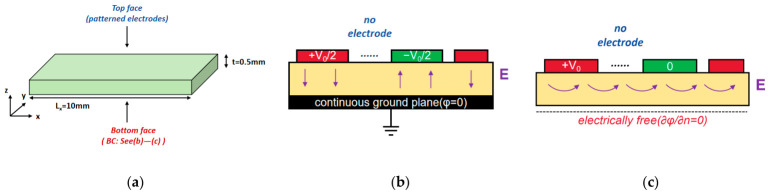
Geometry and boundary configurations. (**a**) Wafer geometry; (**b**) Configuration A—Grounded plane; (**c**) Configuration B/C—Single-sided drive. Wafer geometry and the three bottom-face boundary configurations compared in this work. Configuration A imposes a continuous metallic ground plane on the bottom face and drives the top face symmetrically. Configurations B and C leave the bottom face electrically free; B retains symmetric top drive (with a weak global anchor to remove the additive null mode), while C uses asymmetric drive $(V_{+}=V_{0}$ vs. $V_{-}=0$) so the “0” electrode itself serves as the reference. Configurations B and C produce identical physics (Section 2.4.4); we use C operationally throughout.

We executed the topology-optimization framework in Section 3 across all combinations of five LiNbO_3_ crystal cuts (X, Y, Z, 41°Y, 128°Y), five excitation frequencies in the range 3.30–4.10 MHz (in 0.20 MHz steps), and the two operationally distinct bottom-face configurations illustrated in Figure 1 and introduced in Section 2.4 (Configuration A: grounded plane; Configuration C: single-sided drive in Figure 1). Each (cut, frequency, BC) combination was solved with two independent random initializations of the design field, retaining the higher-purity solution. For comparison, we also computed the bipolar baseline shear purity $\eta_{s}^{bipolar}$ obtained from a simple two-electrode pattern (top half $V_{+}$, bottom half $V_{-}$) without optimization. This yields a complete dataset of 5 × 5 × 2=50 optimized purity values plus 50 corresponding baseline values, totalling 100 measurements. Optimization configuration parameters follow Supplementary Materials, Section S9: $\beta_{0}=4\to \beta_{max}=10$, move limit $\Delta_{0}=0.15$, 120 outer iterations.

#### 4.1.2. Baseline Validation: X-Cut as a Saturated Reference

The X-cut data provide an internal consistency check on the framework. As predicted by the rotated-tensor analysis in Section 2.2—where the dominant element $e{’}_{35}=3.7$ C⋅m^−2^ couples through-thickness $E_{z}$ directly to the shear strain $S_{5}=2\varepsilon_{xz}$—X-cut is naturally suited to the grounded sandwich-electrode geometry of Configuration A.

In summary, under Configuration A, the bipolar baseline already achieves 92.49–97.37% purity across the 3.30–4.10 MHz band, with a peak of 97.37% at 3.70 MHz. Topology optimization yields a peak of 97.49% at the same frequency—a marginal improvement of $\Delta \eta_{s}=+0.12$ percentage points. Across all five frequencies, the mean optimization gain is +0.004 pp. This places X-cut firmly in what we term Regime II of the optimization-utility taxonomy (Section 4.5): a near-optimal baseline that the optimizer cannot meaningfully improve.

The validation message is twofold. First, the framework correctly identifies X-cut as intrinsically a high-purity shear cut requiring no electrode patterning beyond the simple bipolar geometry—recovering a result well-known in the LiNbO_3_ literature [50]. Second, the framework’s quantitative agreement with the bipolar baseline at this physically extremal point confirms the absence of systematic bias in the optimizer, the FE discretization, the rotation tensor implementation, or the adjoint sensitivity formulation. Were any of these to contain errors, the X-cut purity would not converge to the published reference value of ∼97%.

#### 4.1.3. Numerical Validation: Mesh Convergence and Initialization Robustness

**Figure 2:**
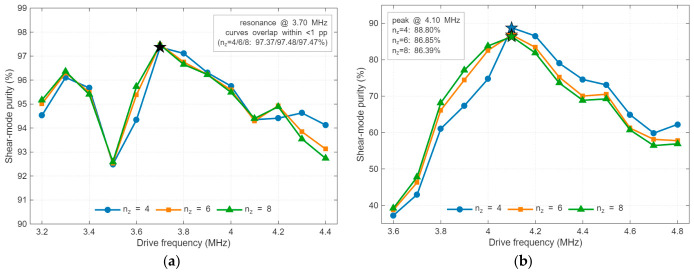
Mesh-convergence of shear-mode purity versus drive frequency for n_z = 4, 6, 8: (**a**) X-cut, grounded—mesh-independent, resonance at 3.70 MHz; (**b**) 128°Y, single-sided—optimal frequency fixed at 4.10 MHz, peak purity converged for n_z ≥ 6.

A through-thickness mesh-convergence study ($n_{z}$ = 4, 6, 8) was performed on the deterministic bipolar baseline, sweeping frequency to control for the mesh-dependent resonance shift (Figure 2). For the X-cut, the purity is essentially mesh-independent (curves overlap within <1 pp; resonance fixed at 3.70 MHz; drift 0.11 pp from n_z = 4 to 8). For 128°Y, the optimal frequency is likewise mesh-independent (4.10 MHz) and the peak purity converges for n_z ≥ 6 (−0.46 pp from $n_{z}$ = 6 to 8); the coarse $n_{z}$ = 4 mesh mildly and monotonically overestimates the near-resonance purity by ≤2.4 pp, without affecting the comparative conclusions. Optimizer robustness was assessed with eight independent random initializations for two representative cases: 41°Y @ 3.50 MHz (grounded) gave mean 76.8%, s.d. 3.4 pp, best 80.69% (equal to the reported value), and 128°Y @ 4.10 MHz (single-sided) gave mean 91.5%, s.d. 1.17 pp, best 93.03% (the reported 92.75% lies within this range), confirming that the best-of-multistart values are reproducible.

#### 4.1.4. Convergence Behavior

**Figure 3:**
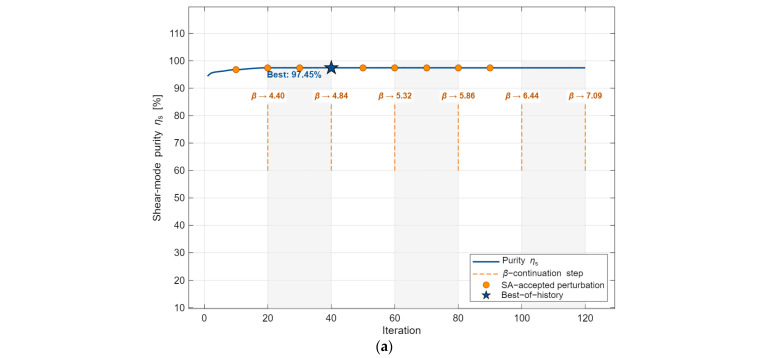

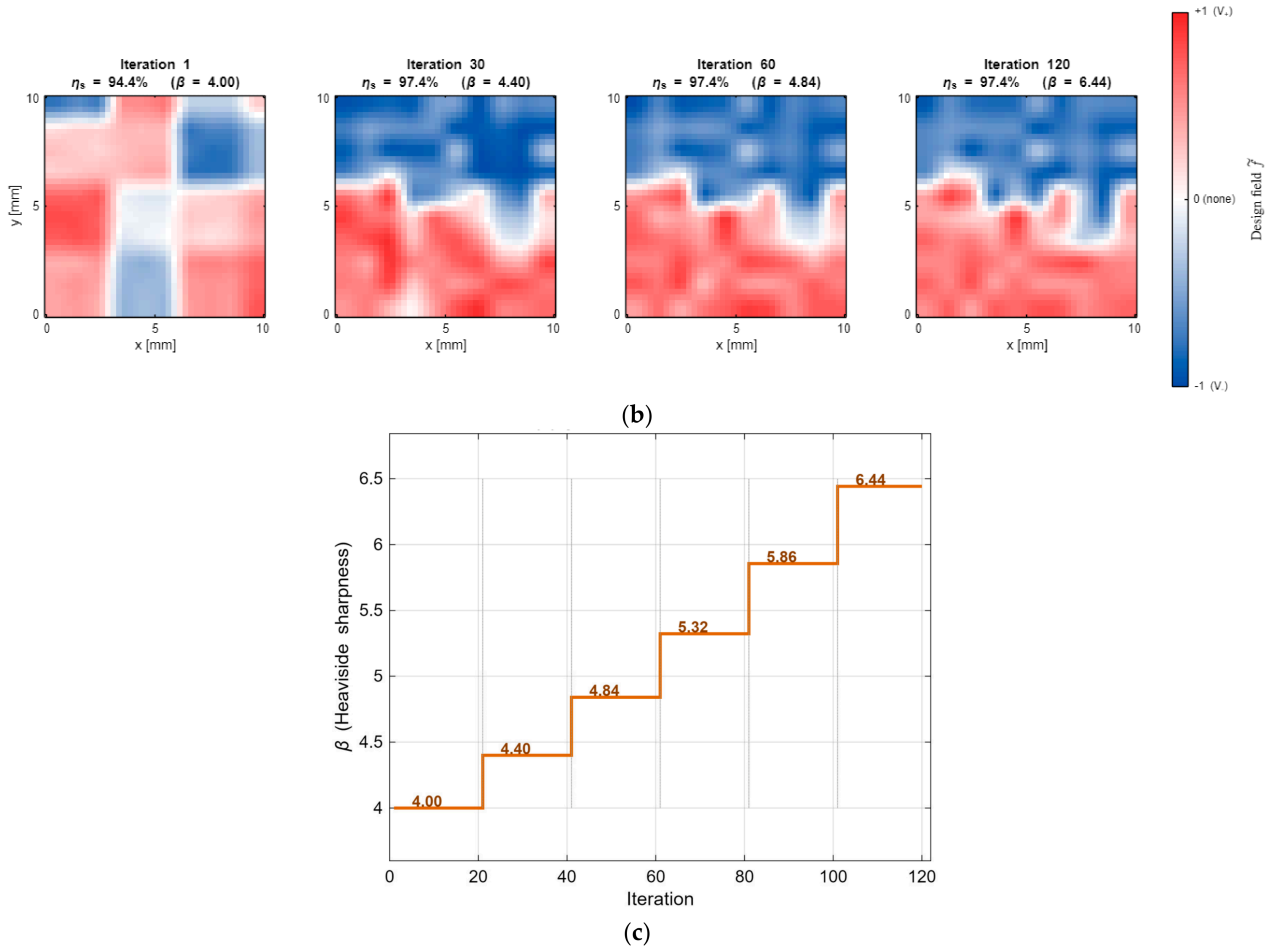
Representative convergence behavior. (**a**) Optimization trajectory: X-cut @ 3.70 MHz, Configuration A; (**b**) Design-field snapshots; (**c**) β-continuation schedule. Representative optimization trajectory for X-cut at 3.70 MHz under Configuration A. The objective J (purity minus regularization penalties) increases steadily through 120 iterations, with characteristic step-down events at β-continuation points (every 20 iterations) where the Heaviside projection sharpens. Simulated-annealing perturbations (every 10 iterations) provide local-minimum escape. The final electrode pattern is fully resolved into discrete tri-state regions ($+V_{0}$, 0, no electrode).

As shown in Figure 3, for the 100 (cut, frequency, BC) optimization runs in this study, convergence to a stable local optimum was achieved in all cases within 120 iterations. The largest jumps in J occurred at β-continuation events when the Heaviside projection sharpened, occasionally producing temporary decreases in J, which were subsequently recovered by the projected-gradient updates. No runs exhibited divergence of $\|\varphi {\|}_{\infty }$ above the BC-dependent thresholds $\varphi_{max}^{thr}$ (Supplementary Materials, S9), confirming the appropriateness of the threshold choice.

### 4.2. Largest Optimization Gains: 41°Y- and 128°Y-Cuts

The most dramatic optimization gains occur for cuts where the bipolar baseline is geometrically mismatched to the dominant coupling pathway. We discuss 41°Y-cut and 128°Y-cut in detail.

#### 4.2.1. 41°Y-Cut: Strong $E_{z}$-Shear Coupling, Mismatched Baseline

For 41°Y-cut, the rotated-tensor analysis predicts strong $E_{z}\to S_{4}$ coupling through ${e^{\prime }}_{34}=4.31$ C⋅m^−2^, the largest single shear-coupling element across the five cuts considered.

The cut-dependent boundary-condition preference follows directly from the rotated piezoelectric stress tensor: orientations with strong shear coupling to the vertical field (large ${e^{\prime }}_{35}$, ${e^{\prime }}_{34}$; e.g., 128°Y) favor the single-sided drive that maximizes vertical-field asymmetry, whereas orientations coupling primarily through in-plane fields favor the grounded configuration. This coefficient-level view accounts both for the observed ranking of cuts and for the saturated Z-cut counterexample, in which no electrode pattern can generate the required shear coupling.

However, the symmetric bipolar pattern $\left(+V_{0}/2,-V_{0}/2\right)$ produces $E_{z}$ fields of opposite sign in the two electrode halves, which interfere destructively with the $S_{4}$ mode shape. Topology optimization has full freedom to select non-bipolar geometries that align constructively with $S_{4}$, and it does so to substantial effect under Configuration A, as shown in Table 1.

The optimization contributes 14.8–28.8 percentage points across the lower three frequencies, with the largest single gain (+28.81 pp) at 3.50 MHz. At higher frequencies (3.90, 4.10 MHz), the bipolar baseline approaches its physical ceiling and improvement saturates. This places 41°Y-cut in Regime I at low frequencies (mismatched baseline) and crosses into Regime II at high frequencies (near-optimal baseline)—illustrating that the regime classification is itself frequency-dependent within a single cut.

#### 4.2.2. 128°Y-Cut: Mode-Selective Bias

128°Y-cut shows a similar but less dramatic pattern. Its mixed coupling pathway (with both through-thickness and in-plane $e^{\prime }$ entries non-trivial) means the bipolar baseline mismatches the dominant mode less catastrophically. Under Configuration A, low-frequency baseline values of 33.80–48.34% improve to 55.05–63.21% under optimization. Under Configuration C, however, the high-frequency optimized purity reaches 92.75% at 4.10 MHz, the cut’s overall maximum across both BCs.

Across all five frequencies, the mean optimization gain over the corresponding baseline is +13.5 pp for 41°Y-cut and +9.4 pp for 128°Y-cut—substantially exceeding the 0.0 pp gain observed for X-cut, and confirming the principle that topology optimization is most beneficial precisely for cuts where the simple bipolar configuration is poorly matched to the dominant coupling pathway.

#### 4.2.3. Optimized Electrode Patterns

**Figure 4:**
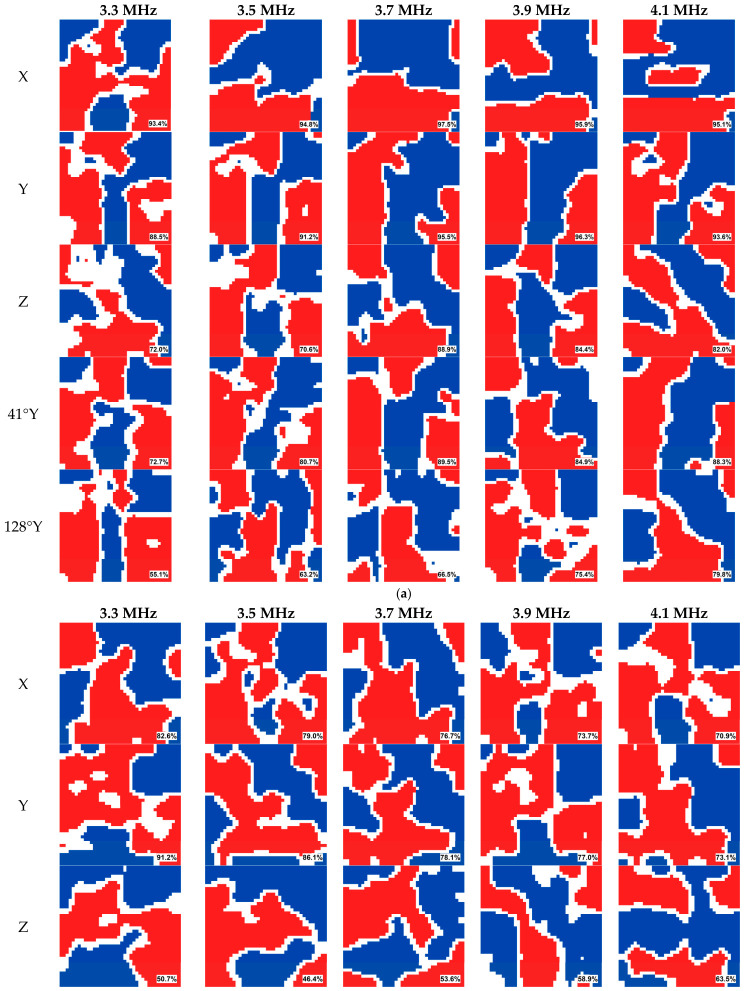

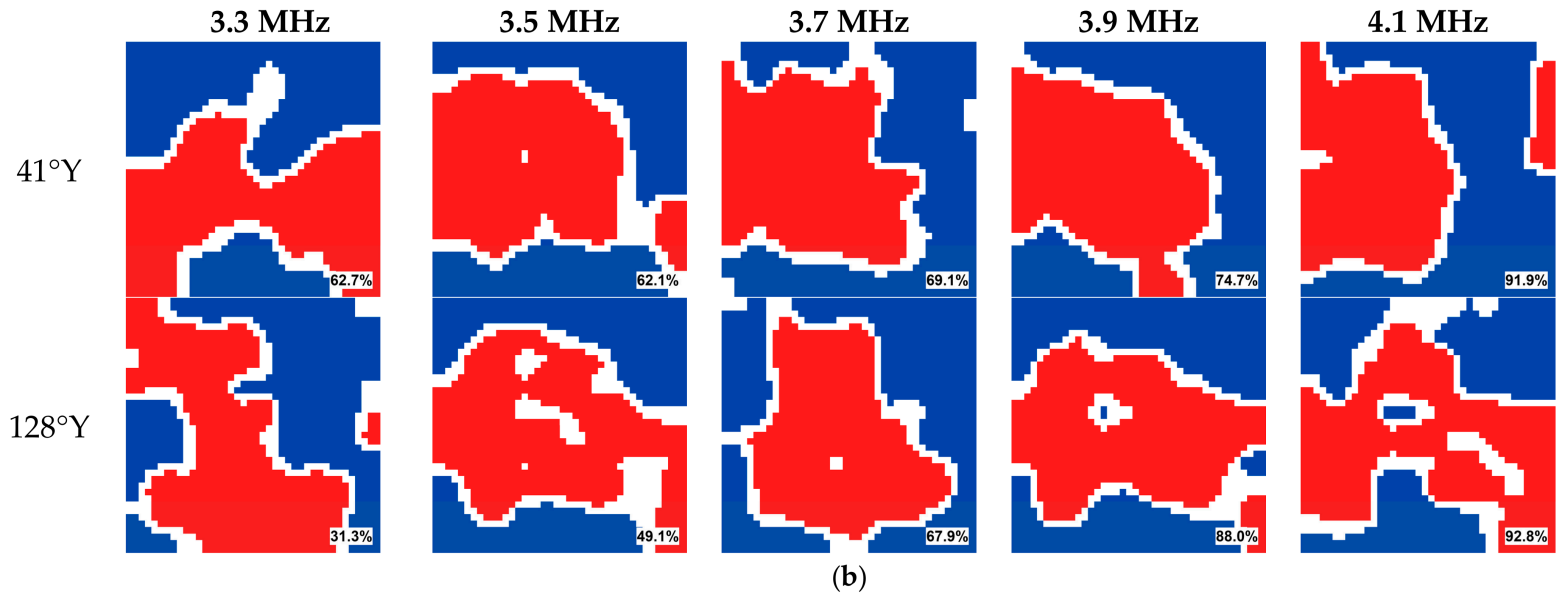
Optimized electrode patterns for all cuts and frequencies. Optimized tri-state electrode patterns for all five crystal cuts (rows) at five excitation frequencies (columns). (**a**) Configuration A: grounded bottom plane. (**b**) Configuration C: single-sided drive. Red regions are $V_{+}$ electrodes, blue regions are $V_{-}$ (**a**) or 0 (**b**), and white regions are unelectroded. The diversity of patterns reflects the strongly cut- and frequency-dependent nature of the optimum. Note the qualitatively different topologies: X-cut and 41°Y-cut typically produce smooth bipolar-like patterns under Configuration A (consistent with the strong $E_{z}$-shear coupling), while 128°Y-cut and Y-cut at higher frequencies under Configuration C produce more elaborate fragmented patterns.

The patterns in Figure 4 visually reinforce the cut-dependent character of the optimum. For X-cut and 41°Y-cut under Configuration A, the optimized geometry resembles a smooth bipolar partition—slightly distorted from the geometric baseline but topologically similar. For 128°Y-cut under Configuration C, the patterns develop richer multi-domain structures with interleaved electrode regions, qualitatively reminiscent of interdigital transducer (IDT) geometries, indicating that the optimizer is leveraging in-plane field components that the bipolar baseline cannot produce. The coarse 10 × 10 design grid enforces a minimum feature size of 1 mm; even the fragmented 128°Y (Configuration C) patterns therefore respect a feature scale several orders of magnitude larger than standard photolithographic limits and are readily manufacturable, including by low-cost patterning such as screen printing or laser ablation.

### 4.3. Cut- and Frequency-Dependent Optimal Bottom-Face Boundary

**Figure 5:**
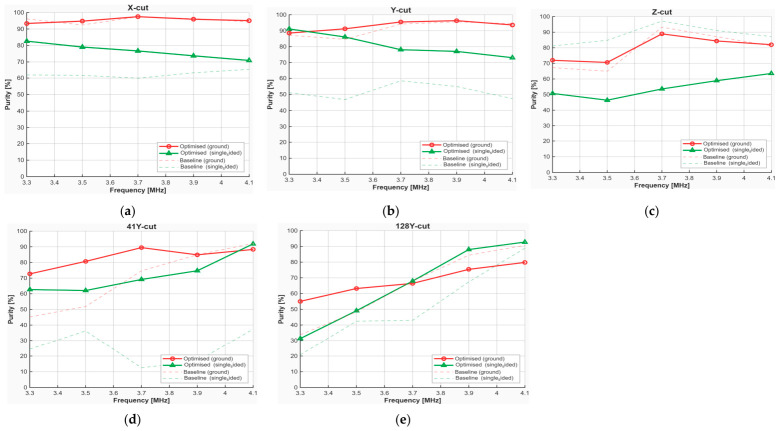
Purity vs. frequency, both configurations, all cuts. (**a**) Purity vs. Frequency (X); (**b**) Purity vs. Frequency (Y); (**c**) Purity vs. Frequency (Z); (**d**) Purity vs. Frequency (41°Y); (**e**) Purity vs. Frequency (128°Y). Shear-mode purity versus excitation frequency for all five crystal cuts and both bottom-face configurations. Solid lines: topology-optimized purity. Dashed lines: simple bipolar baseline. Configuration A (red): grounded bottom plane with symmetric top drive. Configuration C (green): single-sided drive with floating bottom. The qualitative differences across cuts illustrate the central finding of this work: there is no universal optimum—the best configuration depends on both crystal cut and operating frequency.

**Figure 6:**
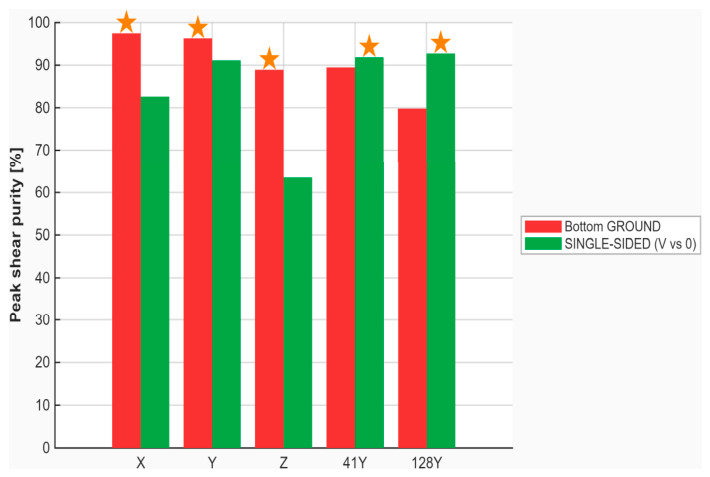
Peak purity per cut, side-by-side. Peak optimized shear-mode purity per crystal cut, comparing the two bottom-face configurations. Star annotations indicate the winning configuration. X-cut and Y-cut (whose dominant coupling involves $E_{z}$, while 128°Y couples predominantly through in-plane fields) attain their highest purity under the grounded configuration; 41°Y-cut shows a near-tie with marginal preference for single-sided; 128°Y-cut exhibits strong preference for single-sided drive.

**Figure 7:**
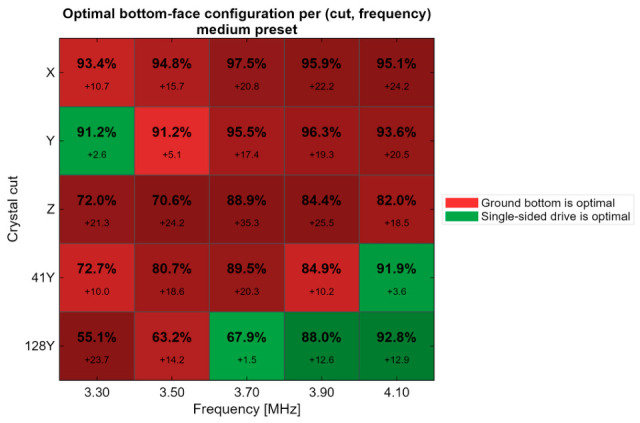
Optimal BC at every (cut, frequency) cell. Optimal bottom-face configuration at every (cut, frequency) cell. Red cells: grounded preferred; green cells: single-sided preferred. Color intensity encodes the magnitude of advantage over the alternative configuration. Numerical labels show the peak optimized purity (large) and the advantage in percentage points (small). Of the 25 cells, 20 favor the grounded configuration and 5 favor single-sided. The frequency-dependent crossover for several cuts is evident—neither configuration is uniformly best across the entire (cut, frequency) plane.

A central finding of this work is that no single bottom-face configuration is uniformly optimal across crystal cuts. Figure 5 displays purity-versus-frequency curves under both configurations for each of the five cuts; Figure 6 summarizes peak purity per cut; and Figure 7 visualizes the winner at every (cut, frequency) cell on a 5 × 5 grid.

Three patterns emerge from Figure 5 and Figure 6:

(i) Cuts whose shear couples to the thickness field, with little parasitic longitudinal coupling, prefer a grounded bottom. X-cut (peak 97.49% in Configuration A) and Y-cut (peak 96.31% in Configuration A) both attain their highest purity under grounded bottom across most frequencies. The advantage over single-sided drive is 14.86 pp for X-cut and 5.16 pp for Y-cut at their respective peaks.
(ii) Cuts with mixed coupling pathways favor single-sided drive at higher frequencies. 128°Y-cut peaks at 92.75% under Configuration C at 4.10 MHz, exceeding the corresponding grounded peak (79.81%) by 12.94 pp. The crossover behavior is clearly visible in the 128°Y panel of Figure 5: at 3.30 MHz, the grounded configuration leads (55.05% vs. 31.34%), but the single-sided curve ascends more steeply with frequency and overtakes near 3.70 MHz.
(iii) Y-cut shows mixed behavior with frequency-dependent crossing. At 3.30 MHz, single-sided drive achieves 91.15% versus 88.53% for grounded (a 2.62 pp single-sided edge). At higher frequencies, the grounded configuration is preferred. This intermediate behavior reflects the mixed coupling pathway of Y-cut, with ${e^{\prime }}_{14}=3.7$ (driving $S_{4}$ from $E_{x}$) and ${e^{\prime }}_{32}=2.5$ (driving longitudinal $S_{2}$ from $E_{z}$) contributing comparable magnitudes (Equation (5)).

The 5 × 5 grid in Figure 7 makes the BC selection rule explicit: out of 25 (cut, frequency) cells, 20 favor grounded and 5 favor single-sided. No frequency, and no cut, is uniformly best for either configuration. A practical implication is that the optimal device geometry should be selected jointly with the operating frequency rather than fixed at the design stage. This conclusion would not have been visible from the existing literature, which typically restricts itself to one BC choice (almost always grounded) made at the device-design level.

Physical origin of the cut-dependent electrode morphology. The optimal pattern for each cut is dictated by which electric-field component carries the shear coupling in the rotated tensor e′ (Equation (5)). Two ratios govern the behavior: the shear coupling to the thickness field, max$\left|e_{3j}^{\prime }\right|$ (j = 4, 5, 6), relative to the shear coupling to the in-plane field, max$\left|e_{1j}^{\prime }\right|$, $\left|e_{2j}^{\prime }\right|$; and the parasitic coupling of the thickness field to longitudinal strain, $\left|e_{31}^{\prime }\right|$, $\left|e_{32}^{\prime }\right|$, $\left|e_{33}^{\prime }\right|$.

For X-cut, the thickness field drives shear with $e_{35}^{\prime }$ = 3.70 cm^−2^, while its longitudinal coupling vanishes identically ($e_{31}^{\prime }$ = $e_{32}^{\prime }$ = $e_{33}^{\prime }$ = 0). A grounded bottom plane, which establishes a nearly uniform $E_{z}$, therefore excites shear almost exclusively; the optimizer has little to improve upon, and the converged pattern remains a simple, large-area bipolar layout (Figure 4). Y-cut shares the same shear pathway ($e_{34}^{\prime }$ = 3.70) but the thickness field also drives longitudinal strain ($e_{31}^{\prime }$ = $e_{33}^{\prime }$ = 2.5), which competes with it and explains both its slightly lower purity and its frequency-dependent crossover.

For 41°Y and 128°Y, the situation is reversed: shear couples predominantly to the in-plane field (max$\left|e_{1j}^{\prime }\right|$ = 4.31 and 4.25 cm^−2^, respectively), whereas the thickness pathway is weak (1.68 and 0.88). In-plane field components are generated only at electrode edges, so the optimizer maximizes edge density, producing the fragmented, interdigital-like morphologies observed for 128°Y (Figure 4). Moreover, 128°Y has the strongest parasitic thickness-to-longitudinal coupling of all cuts ($e_{33}^{\prime }$ = 3.51), so a grounded plane actively injects longitudinal energy; removing it—i.e., the single-sided configuration—suppresses this channel, which is why 128°Y prefers single-sided drive by 12.94 pp.

Z-cut is the limiting case of this logic: its thickness field produces no shear at all ($e_{34}^{\prime }$ = $e_{35}^{\prime }$ = $e_{36}^{\prime }$ = 0), so shear can only be generated by in-plane fields at electrode edges. The bipolar pattern, with its single central boundary, is already the optimal edge configuration at this scale; further fragmentation merely dilutes the coherent edge field, which is the physical reason topology optimization degrades purity for this cut (Section 4.4).

### 4.4. The Z-Cut Counterexample: Limits of Topology Optimization

A particularly instructive case arises for Z-cut. The rotated-tensor analysis (Section 2.2) predicts that Z-cut cannot generate shear from $E_{z}$ because the third row of $e^{\prime }$ is (0.23,0.23,1.33,0,0,0), with all shear components identically zero. Shear excitation must therefore rely entirely on lateral $E_{x},E_{y}$ fields, which are most efficiently produced under floating-bottom (single-sided) drive.

We confirm this prediction quantitatively for the simple bipolar baseline. Under Configuration C at 3.70 MHz, the bipolar pattern achieves $\eta_{s}^{bipolar}=97.15\%$—the highest baseline value of any (cut, frequency, BC) combination in the entire study. By contrast, the same bipolar pattern under Configuration A reaches only 93.24%. The 3.91 pp advantage of Configuration C over A on the baseline confirms the lateral-field hypothesis.

#### 4.4.1. The Counterintuitive Finding

Counterintuitively, however, the topology optimizer fails to improve upon—and substantially degrades—the Z-cut baseline under Configuration C, as shown in Table 2.

**Figure 8:**
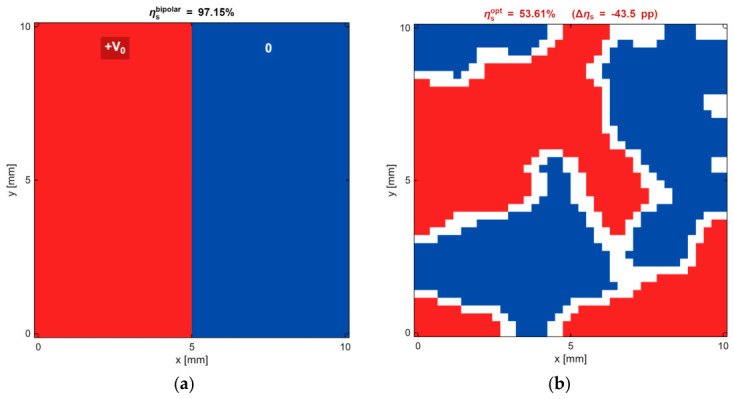

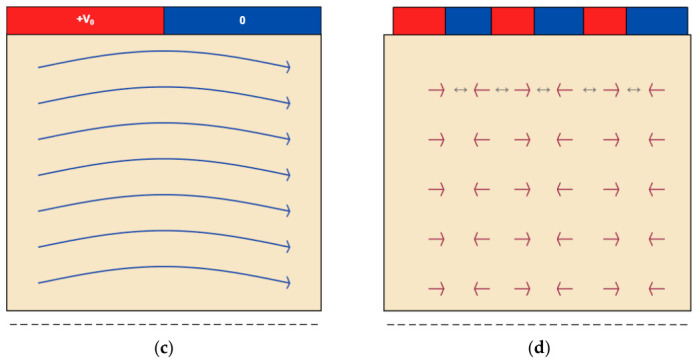
Z-cut counterexample: baseline vs. optimized electrode patterns. The Z-cut counterexample. (**a**) The simple bipolar baseline electrode pattern under single-sided drive—top half at $V_{+}$, bottom half at 0. (**b**) The corresponding optimization result, which deviates substantially from the bipolar partition by introducing fragmented sub-domains. (**c**) Lateral field magnitude $|E_{x}{|}^{2}+|E_{y}{|}^{2}$ in a through-thickness slice for the baseline, showing strong, well-aligned fields that efficiently excite the in-plane-only Z-cut shear coupling. (**d**) The same field for the optimized pattern, showing fragmented, less coherent lateral fields that destructively interfere. The optimizer is constrained by area-balance soft penalties to deviate from the perfect bipolar partition, and these deviations cost more in purity than they gain in regularization compliance.

The optimized purity is 23–44 percentage points *below* the baseline at every frequency. As shown in Figure 8, we observed this same pattern (sign and approximate magnitude) across both random initializations at every frequency, ruling out a multistart-coverage artifact.

#### 4.4.2. Mechanism: Soft Constraints Versus Saturated Baselines

The cause is the soft-penalty constraint structure of the optimization objective (Equation (14)). The bipolar baseline corresponds to electrode area fractions $w^{+}=w^{-}=0.5$, which the soft penalty $\lambda_{A}[(w^{+}-V_{T}{)}^{2}+(w^{-}-V_{T}{)}^{2}]$ pushes towards $V_{T}=0.45$. At the bipolar baseline, however, the gradient of $\eta_{s}$ is approximately zero (the baseline already saturates the available purity), and small movements toward $V_{T}=0.45$ can nudge the optimizer onto a fundamentally different—and worse—basin of attraction. The optimized solutions exhibit larger fragmented patterns (rather than the clean bipolar partition) that disrupt the lateral $E_{x},E_{y}$ flow required for in-plane-only shear excitation.

This Z-cut behavior is not an artifact of insufficient multistart coverage, the limited iteration count, or an implementation bug. It reflects a fundamental property of the soft-penalty optimization framework: when the objective is strongly aligned with a pre-existing geometric symmetry, regularization terms that pull the design away from that symmetry will dominate. We classify this regime as Regime III (optimally matched baseline) in Section 4.5.

#### 4.4.3. Practical Implication

The practical implication is significant: an “optimised” Z-cut transducer would actually exhibit lower shear purity than a trivially fabricated bipolar electrode. When designing a Z-cut shear transducer for practical use, the simple bipolar pattern is the correct geometry, and topology optimization provides no benefit. This finding generalizes that practitioners should always cross-check optimized designs against the bipolar baseline, since the numerical optimum is not necessarily the global optimum when soft constraints are active.

This counterexample is, in our view, one of the more valuable findings of this study—not because we have failed to optimize Z-cut, but because we have characterized when topology optimization is and is not the right tool.

### 4.5. Three Optimization Regimes and Practical Design Recommendations

Unless stated otherwise, all recommendations below are made with respect to a single objective—maximum shear-mode purity—rather than displacement amplitude or ease of fabrication; the one exception, where a fabrication consideration (minimum feature size) motivates the recommendation, is noted explicitly. Synthesizing the results of Section 4.1, Section 4.2, Section 4.3 and Section 4.4, we propose a taxonomy of three optimization regimes that classifies the utility of topology optimization for a given (cut, frequency, BC) combination based on the relationship between the bipolar baseline and the achievable physical optimum.

#### 4.5.1. Regime Definitions

**Figure 9:**
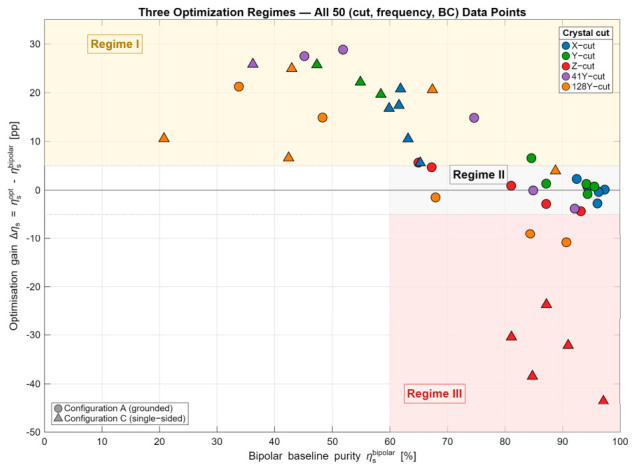
Three optimization regimes (schematic). The three optimization regimes proposed in this work. Each marker represents one (cut, frequency, BC) combination from our study, with horizontal position the bipolar baseline purity and vertical position the optimization gain. Regime I (yellow): mismatched baselines where topology optimization yields large positive gains—exemplified by 41°Y- and 128°Y-cut at low frequencies. Regime II (white): near-optimal baselines where optimization provides marginal benefit—exemplified by X-cut at all frequencies. Regime III (red): optimally matched baselines where topology optimization actively reduces purity due to soft-constraint drag—exemplified by Z-cut under Configuration C. The regime classification provides a priori guidance on whether to pursue topology optimization for a given application.

The three optimization regimes identified above are summarized schematically in Figure 9. This classification organizes the (cut, boundary, frequency) combinations according to how the bipolar baseline relates to the achievable shear-mode purity, and thereby provides an a priori indication of whether topology optimization is expected to yield a meaningful improvement. As shown in Figure 9, the three regimes—mismatched, near-optimal, and optimally matched baselines—correspond to progressively smaller optimization gains, from substantial enhancement in the mismatched regime to negligible or even negative gains once the baseline already approaches the physical purity limit.

Define the baseline–optimization gap

(17) $$\Delta \eta_{s}=\eta_{s}^{opt}-\eta_{s}^{bipolar}$$

The sign and magnitude of $\Delta \eta_{s}$ are governed by two competing effects: (i) pattern enrichment, by which an optimized pattern can exploit fine-grained spatial control of E to better align with the dominant coupling tensor element, producing positive $\Delta \eta_{s}$; and (ii) constraint drag, by which the soft penalties on electrode area balance and pattern smoothness pull the optimum away from a high-purity bipolar configuration, producing negative $\Delta \eta_{s}$.

The interplay between these two mechanisms yields three regimes:

Regime I: Mismatched baseline, large positive $\Delta \eta_{s}$.

When the simple bipolar geometry is poorly aligned with the dominant coupling, the optimizer dramatically improves purity. Examples: 41°Y-cut at 3.30–3.70 MHz (Configuration A), with $\Delta \eta_{s}=+14.8$ to +28.8 pp; 128°Y-cut at 3.30–3.70 MHz (Configuration A), with $\Delta \eta_{s}=+18.5$ to +21.3 pp.

Regime II: Near-optimal baseline, small $\left|\Delta \eta_{s}\right|$.

When the bipolar geometry already matches the dominant coupling pathway, the optimizer has limited room for improvement. Pattern enrichment is marginal; small constraint drag drives $\Delta \eta_{s}$ to near zero or slightly negative. Examples: X-cut at all five frequencies (Configuration A), with $\Delta \eta_{s}$ ranging from −2.75 to +2.29 pp.

Regime III: Optimally matched baseline, large negative $\Delta \eta_{s}$.

When the bipolar geometry is *exceptionally* well-matched, the bipolar baseline can saturate the available purity. In this regime, soft constraints drag the optimizer away from the optimum without the possibility of compensating gain from pattern enrichment. Example: Z-cut under Configuration C at 3.70 MHz, where $\Delta \eta_{s}=-43.54$ pp.

#### 4.5.2. Practical Design Recommendations

The proposed taxonomy provides a practical design guideline. Topology optimization is most beneficial for combinations of crystal cut, frequency, and boundary condition in which the simple bipolar baseline is poorly matched to the dominant piezoelectric coupling pathway. Conversely, when reporting optimized designs, the optimized result should always be compared against the bipolar baseline, because the numerical optimum under soft constraints may be inferior to a simple physically matched geometry.

Specific design recommendations derived from the present dataset are summarized in Table 3.

#### 4.5.3. Comment on the Optimization Algorithm Choice

The Regime III behavior described in Section 4.4 reflects a limitation of the soft-penalty formulation in Equation (14), rather than a fundamental limitation of topology optimization itself. A constrained optimization formulation with hard inequality constraints on purity and equality or inequality constraints on electrode area balance, for example, one solved using the Method of Moving Asymptotes, could in principle eliminate the constraint-drag pathology. In such a formulation, constraints such as $w^{+}$, $w^{-}\leq V_{T}^{max}$ would replace penalties that pull the design toward a prescribed target coverage. The bipolar Z-cut baseline would then remain a feasible optimum, and the optimizer would either retain it or improve upon it, rather than degrading it.

The present work adopts the soft-penalty formulation because of its computational efficiency, compatibility with multistart exploration, and consistency with common practice in the piezoelectric topology-optimization literature. In this context, the Z-cut counterexample should be interpreted as a genuine empirical finding about the behavior of the standard soft-penalty formulation, rather than as an implementation oversight.

## 5. Conclusions and Future Work

### 5.1. Summary of Findings

This study presents a complete topology-optimization framework for ternary single-sided electrode patterns, comprising +V, 0, and electrode-free states, on 10 × 10 × 0.5 mm lithium niobate (LiNbO_3_) wafers. Unlike conventional electrode-design approaches that prescribe the bottom-face electrical condition as a fixed fabrication constraint, the proposed framework treats the bottom-face boundary configuration as an explicit design dimension. The framework integrates complex-Hermitian adjoint sensitivity analysis based on Wirtinger calculus, coarse–fine design-mesh decomposition, and Heaviside projection with β-continuation to obtain manufacturable electrode layouts. A systematic computational study was conducted across five LiNbO_3_ crystal cuts, X, Y, Z, 41°Y, and 128°Y; five excitation frequencies, from 3.30 to 4.10 MHz; and two physically distinct bottom-face configurations, namely, a grounded plane and single-sided drive. Together with the corresponding bipolar baselines, this produced 100 measurements of shear-mode purity and led to three principal findings.

First, the optimal bottom-face boundary condition is both crystal-cut and frequency-dependent, and is governed by the structure of the rotated piezoelectric tensor. Cuts whose dominant shear coupling proceeds via through-thickness $E_{z}$—most strongly, X-cut (${e^{\prime }}_{35}=3.7$ C⋅m^−2^) and 41°Y-cut (${e^{\prime }}_{34}=4.31$ C⋅m^−2^)—achieve their highest purity under the grounded configuration, with peak values of 97.49% and 89.48% respectively. Cuts driven primarily by lateral fields, by contrast, favor the single-sided configuration: 128°Y-cut at 4.10 MHz reaches 92.75% under single-sided drive, exceeding the corresponding grounded peak by 12.94 percentage points. Of 25 (cut, frequency) cells in our design space, 20 favor the grounded configuration and 5 favor single-sided; no configuration is uniformly optimal. This finding is, to our knowledge, the first systematic demonstration that the bottom-face boundary should be treated as an active design variable rather than a fixed fabrication constraint.

Second, topology optimization provides its largest gains when the simple bipolar baseline is mismatched to the dominant electromechanical coupling pathway. Where the simple bipolar geometry is poorly aligned with the dominant coupling pathway—in particular, 41°Y-cut at 3.30–3.70 MHz under grounded drive—the optimizer produces purity improvements of 14.83 to 28.81 percentage points over the bipolar baseline, with the largest single gain (+28.81 pp) at 3.50 MHz. Mean optimization gains across the full frequency range are +13.5 pp for 41°Y-cut and +9.4 pp for 128°Y-cut, dramatically exceeding the +0.004 pp gain observed for the already-saturated X-cut. Topology optimization is therefore most beneficial precisely where it is most needed: for cut-BC combinations where the bipolar baseline is geometrically unsuited to the underlying physics.

Third, topology optimization can become counterproductive when the bipolar baseline is already optimally matched to the underlying physics. For Z-cut LiNbO_3_ under single-sided drive, the simple bipolar electrode pattern produces near-ideal lateral $E_{x}$ and $E_{y}$ fields that directly excite the in-plane shear-coupling pathway of the Z-cut wafer. This configuration achieves a wafer-level baseline purity of 97.15%, the highest baseline value observed among all combinations of crystal cut, frequency, and boundary condition in this study. Under the present soft-penalty formulation, however, the optimizer is unable to retain or improve this saturated baseline. Instead, the area-balance regularization drives the design toward fragmented patterns that reduce shear-mode purity by 23–44 percentage points across the frequency range. This counterexample demonstrates that topology optimization is not universally beneficial and that optimized designs must always be benchmarked against simple, physically motivated baselines.

Based on these findings, the observed behaviors were classified into three regimes: mismatched bipolar baselines, which produce large positive gains after optimization; near-optimal bipolar baselines, which produce negligible or slightly negative gains; and optimally matched bipolar baselines, for which topology optimization may substantially degrade performance. This three-regime taxonomy provides practical guidance for determining when topology optimization is likely to be useful and when a simple electrode geometry should be retained.

### 5.2. Limitations and Future Work

The framework developed in this study intentionally adopts several simplifying assumptions in order to isolate the principal physics of cut-dependent electrode design. These limitations do not undermine the main conclusions, but they identify important directions for extending the present wafer-level model toward full transducer design.

#### 5.2.1. Anisotropic Damping Tensor

Material attenuation is modeled by an isotropic structural loss factor ζ (0.02 for all reported grounded (A) and single-sided (C) results; 0.05 is used only for the alternative floating implementation and as a sensitivity check), approximating the average modal Q-factor. This does not distinguish modal-specific damping; in particular, shear and longitudinal modes in LiNbO_3_ exhibit measurably different Q values. Replacing the scalar ζ with an anisotropic complex-stiffness tensor $c^{\prime *E}=c^{\prime E}(I+i\eta_{m})$ together with a complex permittivity $\varepsilon^{\prime *S}=\varepsilon^{\prime S}(I-i\tan\delta_{e})$ would yield realistic mode-specific Q-factors, at the cost of additional material parameters that must be characterized experimentally for each cut. We expect this extension to refine the absolute amplitude predictions while leaving the cut-dependent boundary findings in Section 4.3 qualitatively intact, since the latter are governed by tensor topology rather than damping detail.

#### 5.2.2. Acoustic Radiation Boundary Conditions

All wafer surfaces are treated as mechanically free in the present model, corresponding to an idealized in vacuo configuration. Practical transducers are typically bonded to a damping backing layer on the bottom and load into a fluid or solid medium on the top; both contribute radiation damping not modeled here. An acoustic-impedance boundary condition $T\cdot n=-i\omega Z_{ac}\tilde{u}$ on the loaded surfaces would model these effects but requires the specification of $Z_{ac}$ for the chosen application. We expect this extension to (a) reduce absolute displacement amplitudes by 1–2 orders of magnitude and (b) lower modal Q-factors and the spectral sharpness of resonance peaks, but (c) leave the shear-purity ratio $\eta_{s}$ approximately invariant, since broadband acoustic dampers attenuate both wave types comparably. Quantifying this expectation is an immediate priority for future work directed at full-transducer modeling.

#### 5.2.3. Multi-Frequency and Bandwidth-Aware Optimization

The present optimization is performed at a single excitation frequency for each problem instance. Many practical transducer applications require high shear-mode purity over a finite bandwidth or across multiple discrete operating frequencies. This is particularly relevant to multi-mode ultrasonic non-destructive testing, where multiple shear-mode families may need to be excited with high purity across a broader frequency range.

Two extensions are especially relevant. The first is a max–min formulation over a discrete frequency set $\left\{f_{1},\ldots ,f_{K}\right\}$, in which the optimizer maximizes the worst-case purity, $\underset{k}{\min}\eta_{s}(f_{k})$. This formulation can be incorporated into the present framework with moderate modification. The second is an integral-band objective over a continuous frequency interval, which would require frequency-domain quadrature and repeated forward solving across the operating band. This approach is more computationally demanding but would provide a more realistic design criterion for broadband transducer applications.

#### 5.2.4. Constrained Formulation via the Method of Moving Asymptotes

The present framework uses soft penalties to control electrode area balance and pattern smoothness, with weighting coefficients $\lambda_{A}$ and $\lambda_{p}$. This formulation is computationally efficient, compatible with multistart exploration, and consistent with common practice in piezoelectric topology optimization. However, it does not guarantee Pareto optimality and can produce the Regime III behavior observed for Z-cut LiNbO_3_, where soft regularization pulls the optimized design away from an already optimal bipolar baseline.

A more rigorous alternative is to formulate the problem using explicit constraints, for example, hard inequality constraints on electrode area and manufacturability, and to solve the resulting constrained optimization problem using the Method of Moving Asymptotes. In such a formulation, constraints such as $w^{+},$ $w^{-}\leq V_{T}^{max}$ could replace penalties that pull the electrode fractions toward a prescribed target value. The bipolar Z-cut baseline would then remain a feasible optimum, and the optimizer would be expected either to retain it or to improve upon it, rather than degrading it. Implementing this constrained formulation on top of the existing complex-Hermitian adjoint pipeline is a natural next step, particularly for the multi-frequency and bandwidth-aware extensions described above.

Shear-mode purity was adopted as the sole objective because it is the primary determinant of mode selectivity and signal-to-noise ratio in shear-wave NDT and sensing, and because it is a dimensionless, scale- and mesh-robust quantity that permits a comparison of cuts and boundary conditions on an equal footing. Displacement amplitude, electromechanical coupling, acoustic power and spurious-mode suppression are also important; they are partially aligned with purity (a pure shear response inherently suppresses unwanted longitudinal content) but not identical to it. A full multi-objective treatment—for example, a purity–amplitude trade-off via the Method of Moving Asymptotes—is a natural extension and is identified here as future work.

Because the thickness-shear resonance scales as $f=\frac{v_{s}}{2t}$ and the design is expressed in dimensionless wafer-fraction coordinates (a 10 × 10 grid over the wafer), the optimized patterns are expected to transfer to geometrically similar wafers of different lateral size at fixed aspect ratio, with absolute frequencies rescaling with thickness. The cut-dependent boundary-condition preferences, which arise from the rotated material tensors rather than from geometry, should persist under moderate changes of dimension and frequency, whereas strongly altered mechanical loading (e.g., heavy backing or clamping) would warrant re-optimization.

### 5.3. Closing Remark

The cut-dependent boundary selection identified in this study points to a broader principle for piezoelectric electrode design in anisotropic non-centrosymmetric crystals: the bottom-face electrical boundary condition is not merely a fabrication detail, but an active design variable whose optimal choice is dictated by the rotated material tensors. This principle is expected to remain valid beyond the specific damping model, surface-loading assumption, optimization algorithm, and frequency specification used in the present work.

This work provides the wafer-level design foundation—validated numerically and internally anchored against the known ~97% X-cut shear purity—on which a forthcoming experimental study will build, in which the optimized single-sided patterns will be fabricated and their measured responses compared with simulation under realistic transducer loading, backing, electrical matching, and application-specific boundary conditions.

Overall, the proposed framework establishes a wafer-level design baseline for high-purity shear-mode LiNbO_3_ transducers. It provides both a computational method for electrode topology optimization and a practical criterion for deciding when such optimization is beneficial. Future work will extend this baseline toward full-transducer modeling, including anisotropic damping, acoustic loading, bandwidth-aware objectives, and constrained optimization formulations, with multi-mode ultrasonic non-destructive testing as an immediate application target.

## Figures and Tables

**Table 1:** Optimization gains for 41°Y-cut (Configuration A).

Frequency	Baseline	Optimized	$\Delta \eta_{s}$
3.30 MHz	45.20%	72.70%	+27.50 pp
3.50 MHz	51.88%	80.69%	+28.81 pp
3.70 MHz	74.65%	89.48%	+14.83 pp
3.90 MHz	84.93%	84.88%	−0.05 pp
4.10 MHz	92.13%	88.32%	−3.81 pp

**Table 2:** Z-cut counterexample under single-sided drive.

Frequency	Baseline(C)	Optimized	$\Delta \eta_{s}$
3.30 MHz	81.17%	50.73%	−30.44 pp
3.50 MHz	84.82%	46.40%	−38.42 pp
3.70 MHz	97.15%	53.61%	−43.54 pp
3.90 MHz	91.03%	58.90%	−32.13 pp
4.10 MHz	87.21%	63.54%	−23.67 pp

**Table 3:** Design recommendations.

Design Goal	Recommended Cut	Recommended BC	Strategy	Expected Purity
Maximum purity, flexible process	X	Grounded	Bipolar (no opt.)	97.5% at 3.7 MHz
Single-sided fabrication	128°Y	Single-sided	Optimized	92.8% at 4.1 MHz
Maximum shear amplitude	41°Y	Grounded	Optimized	89.5% at 3.7 MHz
Z-cut transducer	Z	Single-sided	Bipolar (no opt.)	97.2% at 3.7 MHz

## Data Availability

The original contributions presented in this study are included in the article/Supplementary Material. Further inquiries can be directed to the corresponding author(s).

## Supplementary Materials

The following supporting information can be downloaded at: https://www.mdpi.com/article/10.3390/s26144443/s1, Section S1: Finite-element discretization: mesh, degrees of freedom, and element matrices; Section S2: Degree-of-freedom scaling for ill-conditioned systems; Section S3: Electrode design-field parameterization: tri-state field, density filter, and coarse–fine decomposition; Section S4: Penalty-based boundary enforcement (top- and bottom-face); Section S5: Wirtinger calculus and the complex-transpose distinction; Section S6: Adjoint equation and gradient formula; Section S7: Backward propagation chain; Section S8: Verification by finite differences; Section S9: Optimization algorithm: projected gradient (incl. the divergence threshold, eq. S24), Heaviside continuation, and simulated annealing.
