# Supplementary material for: Cut-Dependent Topology Optimization for Enhancing Shear-Mode Purity in Lithium Niobate Wafers
Source: Sensors (Basel). 2026 Jul 13;26(14):4443. doi: 10.3390/s26144443 (PMC13417318; doi:10.3390/s26144443)
Supplement: Supplementary file 1 [file sensors-26-04443-s001.zip › sensors-4394534-supplementary.pdf]

sensors

MDPI

Supplementary Material

Cut-Dependent Topology Optimization for Enhancing Shear-Mode Purity in Lithium Niobate Wafers

Jun Zhou [1,2,4], Ning Hu[3,*], Weifeng Yuan [2,5], Hui Hu [2], Kaiyan Huang [2,5] and Jishuo Wang [2,5]

[1] School of Mechanical Engineering, Hebei University of Technology, Tianjin 300401, China
[2] Key Laboratory of Testing Technology for Manufacturing Process MOE, Southwest University of Science and Technology, Mianyang 621010, China
[3] School of Mechanical Engineer, Xihua University, Chengdu 610039, China
[4] Sichuan Electronic and Mechanic Vocational College, Mianyang 621023, China
[5] Henan Key Laboratory of Underwater Intelligent Equipment, the 713th Research Institute of China State Shipbuilding Corporation Limited, Zhengzhou 450000, China
* Correspondence: ninghu@xhu.edu.cn (N.H.)

This document provides the detailed numerical implementation referenced from Section 3 of the main text: finite-element discretization, degrees of freedom, and element matrices (S1–S2); the electrode design-field parameterization, including the tri-state Heaviside projection, density filter, and coarse–fine decomposition (S3); the penalty-based enforcement of the three bottom-face configurations (S4); the complex-Hermitian adjoint sensitivity analysis based on Wirtinger calculus (S5–S8); and the optimization algorithm (S9).

S1. Finite-Element Discretization: Mesh, Degrees of Freedom, and Element Matrices

The computational domain Ω is discretized using a structured mesh of $n_x \times n_y \times n_z = 40 \times 40 \times 4$ trilinear hexahedral, or Q_1, elements. This discretization yields $N_{\text{node}} = 41 \times 41 \times 5 = 8{,}405$ nodes and $N_{\text{dof}} = 4N_{\text{node}} = 33{,}620$ total degrees of freedom. At each node, four degrees of freedom are stored in the interleaved order (u_x, u_y, u_z, φ), which improves the spatial locality of the assembled sparse matrices.

The trilinear shape functions are

$$N_a(\xi,\eta,\zeta) = \frac{1}{8}(1+s_a\xi)(1+t_a\eta)(1+r_a\zeta), \quad a = 1,\ldots,8, \tag{S1}$$

with $(s_a, t_a, r_a) \in \{-1,+1\}^3$ the canonical hexahedron node coordinates. The element-level strain–displacement and gradient–potential matrices read

$$\mathbf{B}_u^{(a)} = \begin{pmatrix} \partial_x N_a & 0 & 0 \\ 0 & \partial_y N_a & 0 \\ 0 & 0 & \partial_z N_a \\ 0 & \partial_z N_a & \partial_y N_a \\ \partial_z N_a & 0 & \partial_x N_a \\ \partial_y N_a & \partial_x N_a & 0 \end{pmatrix}, \mathbf{B}_\varphi^{(a)} = \begin{pmatrix} \partial_x N_a \\ \partial_y N_a \\ \partial_z N_a \end{pmatrix} \tag{S2}$$

Each hexahedral element contributes a 32×32 block stiffness matrix and a 32×32 mass matrix. In partitioned form,

$$\mathbf{K}^e = \begin{pmatrix} \mathbf{K}_{uu}^e & \mathbf{K}_{u\varphi}^e \\ (\mathbf{K}_{u\varphi}^e)^\top & -\mathbf{K}_{\varphi\varphi}^e \end{pmatrix}, \quad \mathbf{M}^e = \begin{pmatrix} \mathbf{M}_{uu}^e & \mathbf{0} \\ \mathbf{0} & \mathbf{0} \end{pmatrix} \tag{S3}$$

with the four blocks given by

$$\begin{aligned} \mathbf{K}_{uu}^{e} &= \int_{\Omega_e} \mathbf{B}_u^{\top}\, \mathbf{c}'^{E} \mathbf{B}_u dV \\ \mathbf{K}_{u\varphi}^{e} &= \int_{\Omega_e} \mathbf{B}_u^{\top}\, \mathbf{e}'^{\top} \mathbf{B}_{\varphi} dV \\ \mathbf{K}_{\varphi\varphi}^{e} &= \int_{\Omega_e} \mathbf{B}_{\varphi}^{\top}\, \varepsilon'^{S} \mathbf{B}_{\varphi} dV \\ \mathbf{M}_{uu}^{e} &= \int_{\Omega_e} \rho\, \mathbf{N}_u^{\top} \mathbf{N}_u dV \end{aligned} \tag{S4}$$

The minus sign on the $\mathbf{K}_{\varphi\varphi}$ block arises from the negation of the second weak-form equation in Eq.6 of the main text and ensures that $\mathbf{K}^e$ is real-symmetric. All integrals are evaluated by $2 \times 2 \times 2$ Gauss quadrature.

After standard scatter assembly, the global frequency-domain system is obtained in complex form,

$$\mathbf{A}(\omega)\, \tilde{\mathbf{U}} = \mathbf{F}, \quad \mathbf{A}(\omega) = (1 + i\eta)\mathbf{K} - \omega^2 \mathbf{M} \tag{S5}$$

where $\tilde{\mathbf{U}} \in \mathbb{C}^{N_{\text{dof}}}$ denotes the complex nodal field and $\mathbf{F}$ is the load vector induced by the electrode-imposed boundary conditions.

S2. Degree-of-Freedom Scaling for Ill-Conditioned Systems

A major numerical difficulty in directly solving the assembled system is the severe mismatch between the magnitudes of the mechanical and electric-potential blocks. For the present material and geometric parameters, the diagonal entries associated with the mechanical degrees of freedom are approximately $|K_{uu,ii}| \sim 6 \times 10^7\ \text{N}^{-1}$, whereas those associated with the potential degrees of freedom are approximately $|K_{\varphi\varphi,ii}| \sim 2.4 \times 10^{-13}\ \text{F}^{-1}$. This difference of nearly twenty orders of magnitude makes direct solution of the unscaled system numerically unstable.

To address this conditioning problem, we introduce a diagonal scaling of the electric-potential degrees of freedom,

$$\mathbf{S} = \operatorname{diag}(s_1, \dots, s_{N_{\text{dof}}}), \quad s_k = \begin{cases} 1 & k \in \mathcal{I}_u, \\ s_{\varphi} & k \in \mathcal{I}_{\varphi}, \end{cases} \quad s_{\varphi} = \sqrt{\frac{\text{med}\,|K_{uu,ii}|}{\text{med}\,|K_{\varphi\varphi,ii}|}} \tag{S6}$$

and solve the equivalent symmetrically scaled system.

$$\mathbf{A}_s\, \tilde{\mathbf{U}}^* = \mathbf{F}_s, \quad \mathbf{A}_s = \mathbf{SAS}, \quad \mathbf{F}_s = \mathbf{SF}, \quad \tilde{\mathbf{U}} = \mathbf{S}\tilde{\mathbf{U}}^* \tag{S7}$$

In practice, the scaling factor is typically $s_{\varphi} \approx 1.6 \times 10^{10}$, which reduces the condition number of the global operator by approximately twenty orders of magnitude and makes the system tractable for direct sparse solvers.

S3. Electrode Design-Field Parameterization: Tri-State Field, Density Filter, and Coarse–Fine Decomposition

The top surface carries a ternary electrode pattern. Each design cell can assume one of three physical states: a positive-polarity electrode prescribed at V_+, a negative-polarity electrode prescribed at V_-, or an electrode-free region corresponding to a locally open surface. To make this discrete design space amenable to gradient-based optimization, we encode the electrode pattern through a continuous design field $f(x, y) \in [-1, +1]$ defined on the coarse design grid. Two complementary indicator fields are obtained from the filtered design field through Heaviside projection.

$$w^{\pm}(\tilde{f}) = \frac{1}{2} + \frac{1}{2}\tanh\left[\beta(\pm\tilde{f} - \tau)\right] \tag{S8}$$

Here, $\tilde{f}$ denotes the spatially filtered version of f, $\tau \in (0,1)$ is the half-width of the electrode-free dead zone, and β is the projection sharpness parameter. In this work, we use $\tau = 0.2$ and increased β gradually from $\beta_0 = 4$ to $\beta_{\max} = 10$ through continuation. Under this parameterization, $w^+ \to 1$ identifies regions occupied by the V_+ electrode, $w^- \to 1$ identifies regions occupied by the V_- electrode, and both indicators vanish within the open-circuit dead zone $|\tilde{f}| < \tau$.

To suppress checkerboard artefacts and impose a minimum feature length scale, the fine-scale design field is convolved with a normalized cone, or hat, filter following the standard density-filtering approach.

$$\tilde{f}_k = \frac{\sum_l H_{kl}\, f_l^{\mathrm{f}}}{\sum_l H_{kl}}, \quad H_{kl} = \max(0, r_{\min} - \|\mathbf{x}_k - \mathbf{x}_l\|) \tag{S9}$$

We use a filter radius of $r_{\min} = 1.5$ mm. Because the coarse design grid already enforces large-scale features, the primary role of the density filter in this formulation is to smooth the projected electrode boundaries.

The density filter must be handled carefully during sensitivity backpropagation. The adjoint chain rule requires the normalization by the local filter weight to be applied before multiplication by the transpose of the filter matrix.

$$\frac{\partial J}{\partial f_l^{\mathrm{f}}} = \sum_k H_{kl} \frac{1}{H_{s,k}} \frac{\partial J}{\partial \tilde{f}_k}, \quad H_{s,k} = \sum_l H_{kl} \tag{S10}$$

A common implementation error is to reverse this order. Although the two operations agree in the interior of the domain, where the filter normalization is spatially uniform, they differ near boundaries where the filter kernel is truncated. This error produces systematic gradient inaccuracies near the domain edges. Central finite-difference checks at boundary cells provide a reliable diagnostic for verifying the correct implementation.

For several crystal cuts, particularly the X-cut, the relationship between electrode topology and shear-mode purity is only weakly sensitive to fine-scale geometric details. If the design field is defined directly on the full physical top-face mesh, which contains 1600 design variables in the present 40×40 grid, the optimization tends to generate many near-equivalent local optima distinguished primarily by pixel-scale fragmentation. Such patterns are not practically manufacturable and provide limited physical insight.

We therefore decouple the design and analysis meshes [50]: the design field f^{c} lives on a coarse grid of $n_c \times n_c$ cells (we use $n_c = 10$, totalling 100 design DOFs), and its values on the fine analysis mesh are obtained by bilinear interpolation,

$$\mathbf{f}^{\mathrm{f}} = \mathbf{P}\,\mathbf{f}^{\mathrm{c}}, \quad \mathbf{P} \in \mathbb{R}_{\mathrm{sparse}}^{N_f \times N_c} \tag{S11}$$

where $\mathbf{P}$ has at most four nonzero entries per row, corresponding to the bilinear interpolation weights of the four surrounding coarse cells. The analysis mesh remains at full resolution, so the physical accuracy of the forward solve is unaffected. Sensitivities propagate back to the coarse grid via $\partial J/\partial \mathbf{f}^{\mathrm{c}} = \mathbf{P}^{\top}\, \partial J/\partial \mathbf{f}^{\mathrm{f}}$.

The coarse-fine decomposition serves dual purposes. First, it acts as a strong implicit smoothness constraint, since any pattern expressible on the fine mesh as $\mathbf{P}\mathbf{f}^{\mathrm{c}}$ is necessarily piecewise-bilinear. Second, it reduces the design-space dimensionality by a factor of 16, which dramatically improves the efficiency of multistart exploration and reduces the risk of converging to fragmented local optima.

S4. Penalty-Based Boundary Enforcement (Top- and Bottom-Face)

For each top-face nodal potential degree of freedom k, the corresponding indicator values w_k^+ and w_k^- are obtained by linearly averaging the values of the four adjacent design cells. The penalty contribution is

$$\Delta A_{kk} = \alpha\,[(w_k^+)^p + (w_k^-)^p],\quad \Delta F_k = \alpha\,[(w_k^+)^p\,V_+^* + (w_k^-)^p\,V_-^*] \tag{S12}$$

Here, $V_\pm^* = V_\pm / s_\varphi$ denotes the scaled electrode voltage, $p = 3$ is the SIMP penalization exponent, and $\alpha = 10^4 \cdot \mathrm{med}|K_{uu,ii}|$ is the penalty coefficient. These contributions are added to the global scaled system $(\mathbf{A}_s, \mathbf{F}_s)$. In the limit $w^+ \to 1$ or $w^- \to 1$, the diagonal penalty dominates the original matrix entry by approximately four orders of magnitude, effectively pinning the nodal potential to the prescribed electrode value. Conversely, when $w^+ \to 0$ and $w^- \to 0$, no perturbation is introduced and the potential remains electrically free.

The three boundary configurations introduced in Section 2.4 are implemented as follows.

For **Configuration A**, corresponding to the grounded-plane or sandwich-electrode case, every bottom-face potential degree of freedom receives a strong diagonal penalty with zero load contribution. This enforces $\varphi = 0$ on the bottom face in the same manner as the top-face electrode constraints.

$$\Delta A_{kk} = \alpha,\quad \Delta F_k = 0,\quad k \in \mathcal{I}_\varphi^{\mathrm{bottom}} \tag{S13}$$

The top-face drive is symmetric, with $V_+ = +V_0/2$ and $V_- = -V_0/2$.

For Configuration B, corresponding to a floating bottom face with symmetric top-face drive, no strong bottom-face constraint is imposed. Instead, a weak global anchor of magnitude $\alpha_{\mathrm{weak}} = 10^{-3} \cdot \mathrm{med}|K_{uu,ii}|$ is applied to every bottom-face potential degree of freedom to remove the additive constant null mode without imposing a quantitative grounding condition. Because this anchor is three orders of magnitude weaker than the matrix scale on which it acts, it does not prescribe any local potential value. Rather, it fixes the spatial average sufficiently to obtain a nonsingular numerical system. The top-face drive remains symmetric.

For Configuration C , corresponding to the single-sided drive, no bottom-face penalty is applied. The top-face drive is asymmetric, with $V_+ = V_0$ and $V_- = 0$. In this configuration, the zero-polarity top electrode automatically fixes the absolute reference level of φ, thereby removing the additive null mode without artificial regularization.

The value of α_{weak} in Configuration B must be chosen with care. If it is too small, the system remains numerically singular, typically manifesting as spurious resonances with $\|\varphi\|_\infty \gg V_0$. If it is too large, the weak anchor effectively grounds the bottom face and corrupts the intended floating boundary condition. The selected value places the anchor three orders of magnitude below the typical diagonal scale of the potential block in the scaled matrix. This is sufficient to lift the null mode above the numerical floor while remaining negligible for the determination of the local electric field.

S5. Wirtinger Calculus and the Complex-Transpose Distinction

The state equation (S7) is a linear system in $\tilde{\mathbf{U}}$ with both system matrix and right-hand side depending on the design vector $\mathbf{f}$ through the boundary penalty (Eq. S12):

$$\mathbf{A}(\mathbf{f})\,\tilde{\mathbf{U}} = \mathbf{F}(\mathbf{f}) \tag{S14}$$

Because $\tilde{\mathbf{U}}$ is complex but the design parameters and the objective J are real, we adopt the Wirtinger calculus. For a real-valued functional $J(\tilde{\mathbf{U}}, \tilde{\mathbf{U}}^*)$,

$$dJ = 2\,\mathrm{Re}\left(\frac{\partial J}{\partial \tilde{\mathbf{U}}^*}\,d\tilde{\mathbf{U}}\right) \tag{S15}$$

A subtlety central to our formulation is the distinction between the **complex transpose** $\mathbf{A}^\top$ and the **Hermitian transpose** $\mathbf{A}^H = \overline{\mathbf{A}}^\top$. The Allik–Hughes form ensures $\mathbf{A}^\top = \mathbf{A}$ (complex symmetric), but the structural-damping factor $(1 + i\eta)$ produces a non-zero

imaginary part so that $\mathbf{A}^H \neq \mathbf{A}$. The adjoint equation derived below requires $\mathbf{A}^H, not \mathbf{A}^\top$ — using the latter would yield the wrong gradient direction for any non-zero damping.

S6. Adjoint Equation and Gradient Formula

Differentiating the state equation with respect to the design vector and applying the chain rule yields the standard adjoint sensitivity expression as

$$\frac{dJ}{d\mathbf{f}} = 2\,\mathrm{Re}\left[\lambda^H\left(\frac{\partial \mathbf{F}}{\partial \mathbf{f}} - \frac{\partial \mathbf{A}}{\partial \mathbf{f}}\,\tilde{\mathbf{U}}\right)\right] \tag{S16}$$

where the complex adjoint field λ is obtained by solving the Hermitian-transpose adjoint equation.

$$\mathbf{A}^H\lambda = \frac{\partial J}{\partial \tilde{\mathbf{U}}^*} \tag{S17}$$

For our objective (14) of the main text the adjoint right-hand side restricted to the mechanical block is

$$\frac{\partial J}{\partial \tilde{\mathbf{U}}_m^*} = w_s\,\mathbf{K}_s\,\tilde{\mathbf{u}}_m + w_\ell\,\mathbf{K}_\ell\,\tilde{\mathbf{u}}_m, \quad w_s = \frac{J_\ell}{(J_s + J_\ell)^2}, \quad w_\ell = \frac{-J_s}{(J_s + J_\ell)^2} \tag{S18}$$

with the regularizer contributions added separately downstream (Section S7). A practical aspect of eq.S16: because J is of the quadratic form $\frac{1}{2}\tilde{\mathbf{u}}^H\mathbf{K}\tilde{\mathbf{u}}$, the factor of $\frac{1}{2}$ from the energy definition exactly cancels the factor of 2 from the Wirtinger formula (eq.S15), so the implementation contains no explicit $1/2$ or 2 multiplicative factors.

S7. Backward Propagation Chain

The full sensitivity backward chain — from J to the coarse design vector $\mathbf{f}^{\mathrm{c}}$ — is a sequence of seven linear operations. We list each step, indicating where regularizer terms enter:

Step 1 — Adjoint solve: Solve (S17) for $\lambda \in \mathbb{C}^{N_{\mathrm{dof}}}$.

Step 2 — Sensitivity to nodal indicators: For each top-face φ-DOF k at node n,

$$\frac{\partial J}{\partial w_n^\pm} = \alpha\,p\,(w_n^\pm)^{p-1}\,\mathrm{Re}\left[\overline{\lambda_k}\,(V_\pm^* - \overline{\tilde{U}_k})\right] + \frac{-2\lambda_A(\bar{w}^\pm - V_T)}{N_{\mathrm{cell}}} \tag{S19}$$

Step 3 — Project to design cells: Using the adjoint of the cell-to-node averaging,

$$\frac{\partial J}{\partial w_c^\pm} = \sum_{n\in\mathcal{N}(c)} \frac{1}{|\mathcal{N}(n)|}\,\frac{\partial J}{\partial w_n^\pm} \tag{S20}$$

Step 4 — Through the Heaviside projection:

$$\frac{\partial J}{\partial \tilde{f}_c} = \frac{\partial J}{\partial w_c^+}\,\frac{dw^+}{d\tilde{f}}|_c - \frac{\partial J}{\partial w_c^-}\,\frac{dw^-}{d\tilde{f}}|_c + \frac{2\lambda_P}{N}\,[\Delta_5\,\tilde{f}]_c \tag{S21}$$

where the minus sign on the second term reflects $\partial w^-/\partial(-\tilde{f}) = +(\beta/2)\,\mathrm{sech}^2[\beta(-\tilde{f} - \tau)]$, and the perimeter contribution (last term) follows from Eq.16 of the main text.

Step 5 — Through the density filter: Using the correctly ordered adjoint (Eq. S10).

Step 6 — Through the coarse–fine interpolation:

$$\frac{\partial J}{\partial \mathbf{f}^{\mathrm{c}}} = \mathbf{P}^\top\,\frac{\partial J}{\partial \mathbf{f}^{\mathrm{f}}} \tag{S22}$$

S8. Verification by Finite Differences

The correctness of the entire chain (Eqs.S16–S22) was verified by central finite differences at multiple test cells and step sizes. Eight design cells were selected at random in a smooth interior region of the Heaviside projection (i.e., where $|\tilde{f}| > \tau + 0.1$, avoiding the high-curvature transition band of sech^2), and the adjoint gradient at each cell was compared against $(J(\mathbf{f} + \epsilon\,\mathbf{e}_c) - J(\mathbf{f} - \epsilon\,\mathbf{e}_c))/(2\epsilon)$ for $\epsilon \in \{10^{-5}, 10^{-4}, 10^{-3}\}$. After applying

the correct ordering of H_s in Eq.S10, the forward-difference convention in Eq.15–Eq.16 of the main text, and the conjugate transpose in Eq.S17, agreement was four significant figures across all tested cells and step sizes.

S9. Optimization Algorithm: Projected Gradient (incl. the divergence threshold, Eq.S24), Heaviside Continuation, and Simulated Annealing

We apply a projected gradient ascent on the bound-constrained design vector $\mathbf{f}^{\mathrm{c}} \in [-1,+1]^{N_c}$ with backtracking line search. The basic update is

$$\mathbf{f}^{\mathrm{c}}_{\mathrm{trial}} \leftarrow \mathrm{clip}(\mathbf{f}^{\mathrm{c}} + \Delta\, \nabla_{\mathbf{f}^{\mathrm{c}}} J / \|\nabla\|_{\infty}, [-1,+1]) \tag{S23}$$

with move limit $\Delta = 0.15$ initially. The trial is rejected and the step shortened ($\Delta \leftarrow 0.4\Delta$, up to eight attempts) if either the objective drops by more than 0.01 or the maximum nodal potential $\|\varphi\|_{\infty}$ exceeds a configuration-dependent threshold $\varphi_{\max}^{\mathrm{thr}}$:

$$\varphi_{\max}^{\mathrm{thr}} = \begin{cases} 5V_0 & \mathrm{Configuration A (grounded)} \\ 100V_0 & \mathrm{Configurations B, C (floating/single-sided)} \end{cases} \tag{S24}$$

This BC-dependent threshold reflects the different physical $\|\varphi\|_{\infty}$ ranges of the three configurations. In Configuration A, the bottom-face Dirichlet condition pins one end of the field, so $\|\varphi\|_{\infty}$ is bounded by $V_0/2$ in well-conditioned cases; exceedances above $5V_0$ indicate pseudo-resonance artifacts. In Configurations B and C, no Dirichlet condition pins the bottom, and near a thickness-shear resonance $\|\varphi\|_{\infty}$ can legitimately be amplified by factors of 10–50. The looser threshold used for these configurations prevents the backtracking procedure from rejecting physically valid resonance-enhanced solutions.

To resolve the discrete electrode pattern, the Heaviside sharpness β is increased gradually. Every 20 iterations, $\beta \leftarrow \min(1.1\beta, \beta_{\max})$ and $\Delta \leftarrow 0.75\Delta$. This continuation strategy prevents premature commitment to local optima while ultimately producing a binary-like electrode field at the maximum sharpness $\beta_{\max} = 10$.

Every 10 iterations, a random simulated-annealing perturbation of amplitude $T_t = T_0 e^{-t/\tau_T}$ is attempted, accepted or rejected by the Metropolis criterion, to escape local minima. The same threshold $\varphi_{\max}^{\mathrm{thr}}$ from Eq.S24 is also applied to SA acceptance.
